# Prediction of cell states and key transcription factors of the human cornea through integrated single-cell omics analyses

**DOI:** 10.1093/pnasnexus/pgaf235

**Published:** 2025-07-29

**Authors:** Julian A Arts, Sofia Fallo, Melanie S Florencio, Jos G A Smits, Dulce Lima Cunha, Janou A Y Roubroeks, Mor M Dickman, Vanessa L S LaPointe, Rosemary Yu, Huiqing Zhou

**Affiliations:** Department of Molecular Developmental Biology, Radboud Institute for Molecular Life Sciences (RIMLS), P.O. Box 9101, Nijmegen 6500HB, The Netherlands; Department of Cell Biology-Inspired Tissue Engineering, MERLN Institute for Technology-Inspired Regenerative Medicine, P.O. Box 616, Maastricht 6200MD, The Netherlands; Department of Cell Biology-Inspired Tissue Engineering, MERLN Institute for Technology-Inspired Regenerative Medicine, P.O. Box 616, Maastricht 6200MD, The Netherlands; Department of Molecular Developmental Biology, Radboud Institute for Molecular Life Sciences (RIMLS), P.O. Box 9101, Nijmegen 6500HB, The Netherlands; Department of Human Genetics, Radboud University Medical Center, P.O. Box 9101, Nijmegen 6500HB, The Netherlands; Department of Molecular Developmental Biology, Radboud Institute for Molecular Life Sciences (RIMLS), P.O. Box 9101, Nijmegen 6500HB, The Netherlands; Department of Molecular Developmental Biology, Radboud Institute for Molecular Life Sciences (RIMLS), P.O. Box 9101, Nijmegen 6500HB, The Netherlands; Department of Clinical Genetics, Maastricht University Medical Center+, P.O. Box 5800, Maastricht 6202AZ, The Netherlands; Department of Cell Biology-Inspired Tissue Engineering, MERLN Institute for Technology-Inspired Regenerative Medicine, P.O. Box 616, Maastricht 6200MD, The Netherlands; Department of Ophthalmology, University Medical Center Utrecht, P.O. Box 85500, Utrecht 3508GA, The Netherlands; Department of Cell Biology-Inspired Tissue Engineering, MERLN Institute for Technology-Inspired Regenerative Medicine, P.O. Box 616, Maastricht 6200MD, The Netherlands; Department of Molecular Developmental Biology, Radboud Institute for Molecular Life Sciences (RIMLS), P.O. Box 9101, Nijmegen 6500HB, The Netherlands; Department of Molecular Developmental Biology, Radboud Institute for Molecular Life Sciences (RIMLS), P.O. Box 9101, Nijmegen 6500HB, The Netherlands; Department of Human Genetics, Radboud University Medical Center, P.O. Box 9101, Nijmegen 6500HB, The Netherlands

**Keywords:** scRNA-seq, machine learning, scATAC-seq, gene regulatory networks, corneal biology

## Abstract

The cornea, a transparent tissue composed of multiple layers, allows light to enter the eye. Several single-cell RNA-seq (scRNA-seq) analyses have been performed to explore the cell states and to understand the cellular composition of the human cornea. However, inconsistences in cell state annotations between these studies complicate the application of these findings in corneal studies. To address this, we integrated scRNA-seq data from four published studies and created a human corneal cell state meta-atlas. This meta-atlas was subsequently evaluated in two applications. First, we developed a machine learning pipeline cPredictor, using the human corneal cell state meta-atlas as input, to annotate corneal cell states. We demonstrated the accuracy of cPredictor and its ability to identify novel marker genes and rare cell states in the human cornea. Furthermore, cPredictor revealed the differences of the cell states between pluripotent stem cell-derived corneal organoids and the human cornea. Second, we integrated the scRNA-seq-based cell state meta-atlas with chromatin accessibility data, conducting motif-focused and gene regulatory network analyses. These approaches identified distinct transcription factors (TFs) driving cell states of the human cornea. The novel marker genes and TFs were validated by immunohistochemistry. Overall, this study offers a reliable and accessible reference for profiling corneal cell states, which facilitates future research in cornea development, disease, and regeneration.

Significance Statement This study creates a human corneal cell state meta-atlas that provides a common nomenclature of cells in the human cornea, through integrating multiple single-cell RNA-seq (scRNA-seq) analyses. Using this meta-atlas, we developed a machine learning pipeline, cPredictor, to accurately annotate cell states in corneal studies using scRNA-seq. Additionally, we identified distinct transcription factors driving cell states through integrating the atlas with chromatin accessibility data. This meta-atlas and the computational tool cPredictor enable future research in cornea development, disease, and regeneration.

## Introduction

The cornea, a multilayered tissue, acts as a transparent protective shield located at the front of the eye. It allows light to enter the eye and functions as a focusing unit together with the lens. The tissue layers in the cornea have been extensively studied (1, 2). The innermost layer of the cornea, the endothelium, is made of a thin layer of corneal endothelial cells (CEC). The endothelium maintains liquid homeostasis, important for maintaining correct corneal hydration (3). The corneal stroma, composed mainly of keratocytes that produce extracellular matrix proteins, is anterior to the endothelium and plays a role in maintaining the transparency of the cornea as well as providing biomechanical strength for the cornea (4). The corneal epithelium constitutes the outermost layer of the cornea. Composed of stratified epithelial cells linked together by desmosomes and tight junctions, the corneal epithelium functions as a strong physical barrier (5). Beyond the periphery of the cornea is the nontransparent conjunctiva. The conjunctiva has a role in providing ocular lubrication, as well as protecting the underlying tissues from insults, such as dust and microbes, entering deeper into the cornea (6). Between the corneal epithelium and the nontransparent conjunctiva is the limbus. The limbus contains limbal stem cells (LSCs) that are capable of renewing the corneal epithelium and maintaining corneal regeneration (7–10). The limbus also has a role as a physical barrier, preventing conjunctival (Cj) cells and blood vessels from invading the transparent and avascular cornea (11).

Various cell states in the different layers of the human cornea, including limbal, corneal epithelial, Cj, stromal, immune, and vascular cells, have recently been investigated by several studies using single-cell RNA-seq (scRNA-seq) analyses. Nevertheless, the number of defined cell states, their nomenclature, and the corresponding marker genes do not agree among these studies, likely due to differences in data collection, processing, and performed analyses (12). The inconsistencies between these studies make it challenging to apply these findings in follow-up studies on corneal biology and disease. Therefore, integrating these studies to create a cell state meta-atlas that is comprehensive and can be used as a common reference (13) is warranted. Additionally, most of these studies focus on identifying marker genes for cell states, whereas transcription factors (TFs) that play key roles in determining cell states were not centrally studied. Previous research on LSCs reported a small number of TFs, such as PAX6, TP63, FOXC1, RUNX1, and SMAD3 (14). The key TFs in other corneal cell states remain less explored.

To reliably predict TFs controlling cell states, information on both gene expression (e.g. RNA-seq) and genomic regulatory elements that modulate gene expression needs to be incorporated. Regulatory elements are in accessible chromatin regions that can be detected by technologies such as ATAC-seq analysis (15). TF binding to accessible regions can be predicted by motif analyses on the sequences in these genomic regions. RNA-seq and ATAC-seq data can further be integrated into gene regulatory networks (GRNs) that comprise TFs and their target genes (nodes), as well as the regulatory relationships between TFs and target genes (edges). By capturing the regulatory relationships, causality, and combinatorial interactions of TFs and their target genes, GRNs can enhance the ability to predict TFs that drive cell state differences. Thus, by combining gene expression and accessible chromatin regions detected at the single-cell level through scRNA-seq and scATAC-seq, and constructing single-cell GRNs, key TFs controlling specific cell states can be predicated in tissues containing heterogeneous cell types such as the human cornea. Previously, we developed a computational pipeline, single-cell ANANSE (scANANSE) (16), that combines scRNA-seq and scATAC-seq and generates pseudobulk of these datasets to construct robust GRNs from these otherwise sparse data. scANANSE leverages TF expression, TF binding to anticipated target genes, and the expression of these target genes to build GRNs. scANANSE predicts the importance of a TF represented by an influence score through pairwise comparisons of GRNs of two cell states.

In this study, we integrated four publicly available scRNA-seq datasets of the human adult cornea to create a corneal cell state meta-atlas. This meta-atlas allowed us to annotate rare corneal cell states and identify novel marker genes, which were then experimentally validated. To facilitate its future application, we developed a support vector machine (SVM)-based machine learning prediction pipeline, cPredictor, using this meta-atlas as the reference. As proof-of-principle, we showed that cPredictor could accurately annotate cell states in human corneal scRNA-seq studies on human adult corneas and on induced pluripotent stem cell (iPSC)-derived corneal organoids. Furthermore, integration of this scRNA-seq-based corneal cell state meta-atlas with the human cornea scATAC-seq data enabled us to construct GRNs using scANANSE and to identify key TFs driving various cell states in the human cornea.

## Results

### Data collection and integration of scRNA-seq of human corneal studies

To establish a cell state meta-atlas, we collected four scRNA-seq studies on adult human corneas from which data are currently publicly available (17–20) (Table S1). Two of them were generated from complete corneas (17, 18). One study from Collin et al. (17) used six donor corneas, one specifically for retrieving the limbal ring, one for central cornea, and four complete corneas. Of note, in addition to scRNA-seq, this study also reported single-cell ATAC-seq (scATAC-seq) data that were applied later in this study for analyzing genomic regulatory regions. Another study, from Català et al. (18), used eight donor corneas, and two of them specifically used the limbal ring. The third study, from Gautam et al. (19), extracted cells from three whole eyes, of which the cornea data were included in this study. Additionally, we collected data from the study of Li et al. (20) who isolated cells from the limbus of four donor corneas by removing the central cornea and superficial layers. In these four studies, different corneal sample disaggregation methods and data pipelines were used (Table S1), which inevitably gave rise to different numbers and types of corneal cell states (12).

**Fig. 1.**
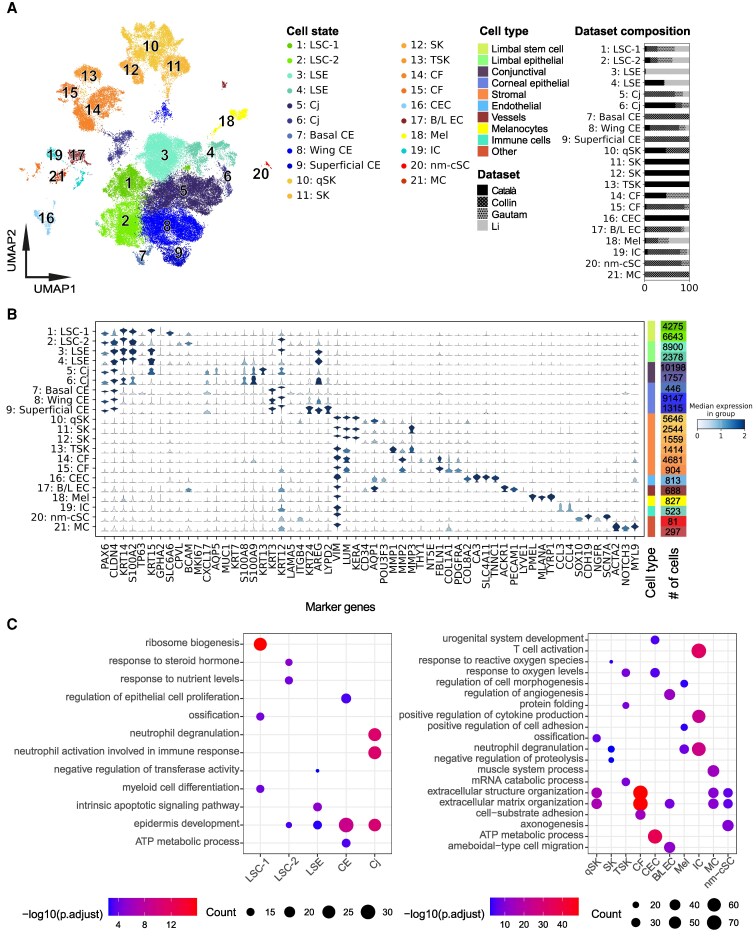
Integration, identification, and characterization of cell states in the human cornea. A) Uniform manifold approximation and projection (UMAP) of integrated and annotated cell states. Colors indicate overall cell type, and subcolors define the respective cell states. The bar plots on the right depict dataset contributions for each cell state. B) Violin plot of the literature annotated marker gene expression across integrated cell states. The height of the violin plot indicates the expression level of the markers, and the color indicates the median score for all cells in that specific cell state. C) GO-term enrichment of selected cell states on all cluster markers identified with Seurat. Names of cell states (A–C): LSC-1, limbal stem cells 1; LSC-2, limbal stem cells 2; LSE, limbal suprabasal epithelium; CE, central epithelium; Cj, conjunctiva; qSK, quiescent stromal keratocytes; SK, stromal keratocytes; TSK, transitioning stromal keratocytes; CF, corneal fibroblasts; CEC, corneal endothelial cells; B/L EC, blood and lymph endothelial cells; Mel, melanocytes; IC, immune cells; nm-cSC, nonmyelinating corneal Schwann cell; MC, mural cell.

To perform consistent analyses, we started with raw sequencing reads of all four studies. We performed data preprocessing, quality control, and doublet removal on all datasets and subsequently used single-cell variational inference (scVI) (21) to integrate the data. scVI is currently considered one of the best integration tools for nonuniformly labeled datasets and is likely to maintain the biological relevance of cell states while minimizing batch effects (22). Next, we applied unbiased Leiden clustering (23) to group cells based on their gene expression profiles (Fig. 1A), identified highly variable genes (HVGs) of each group, and annotated cell states with well-known markers among HVGs (Fig. 1B). This clustering analysis resulted in a total of 21 distinct cell states, with a subdivision into two major branches: 9 limbal/corneal epithelial-related clusters and 12 nonepithelial diverse cell states including stromal cells and immune-related cells (Fig. 1A). Notably, our data integration revealed retrieved cell state differences across studies. For example, cells from cluster 3, cells of limbal suprabasal epithelium (LSE), were mainly from the study of Li et al. (20); most central epithelial (CE) cells, including clusters 7 (basal) and 9 (superficial), were from Collin et al. (17), and clusters 11 and 12, stromal keratocytes (SKs), were largely from Català et al. (18) (Fig. 1A). In addition to scVI, we also used Harmony for data integration (24, 25). In general, Harmony integration revealed similar cell states, as compared to scVI, showing the two major branches. However, individual cell states within each branch were less distinct (Fig. S1A and B), which is consistent with a previous benchmarking study comparing the two methods. We therefore continued downstream analysis using the scVI integrated data.

The branch of nine limbal/corneal epithelial-related clusters expressed *PAX6* (Fig. 1B), a marker for limbal and corneal epithelial cells, and *CLDN4*. This branch contained the largest number of cells, 45.059 out of 65.036 total. Although our nomenclature of cell states was apparently different from previous studies, our annotations of these cell states in the integrated meta-atlas were in general consistent with previous individual studies. For instance, corneal limbal cells from the study of Català et al. (18) and limbal progenitor cells from Collin et al. (17) were not annotated consistently in the two studies but clearly clustered together as a single LSC state in our cell state meta-atlas (Figs. S1C and S2).

The branch represented by 12 nonepithelial cell states expressed *VIM* and consisted of nonepithelial cells, such as stromal cells, fibroblasts, and immune cells (IC). The immune-related cluster was consistent with IC from previous studies (17, 19). However, cells in stroma were annotated differently in previous studies. Specifically, cells classified as fibroblasts in the study by Gautam et al. (19) and SK in the study of Català et al. (18) grouped together, and they were indistinguishable as corneal fibroblasts (CFs) in our meta-atlas. Likewise, corneal stromal cells from Collin et al. (17) and SK from Català et al. (18) clustered together and were annotated as quiescent stromal keratocytes (qSKs). Notably, we identified a previously unidentified cell state showing distinct markers for nonmyelinating corneal Schwann cells (nm-cSCs). They contained a small number of cells (<5%), previously identified as fibroblasts, melanocytes, and corneal endothelial cells (CEC) from individual studies (Figs. S1 and S2). These cells were not identified as nm-cSCs, likely due to their low number in individual studies, highlighting the power of integrating the studies.

### Distinct and shared marker genes in the cell state meta-atlas of the human cornea

**Fig. 2.**
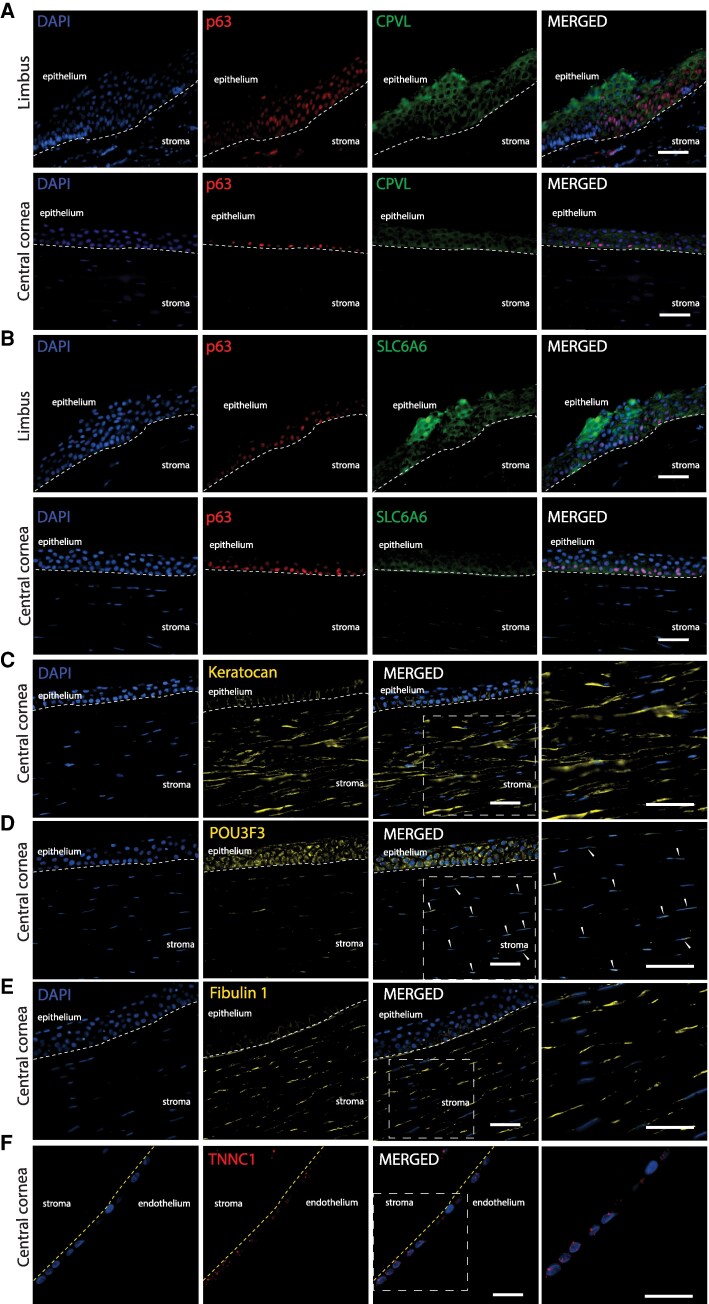
Immunofluorescence of novel markers in the human cornea. A), B) Costaining of CPVL and SLC6A6 (green) with p63 (ΔNp63) (red) in limbus and central cornea. Staining of corneal stroma marker keratocan (C), POU3F3 protein (D), and fibulin-1 (E). F) Staining of TNNC1 in corneal endothelium. Arrowheads depict cells in the stroma with nuclear expression of POU3F3. Dotted lines indicate borders between stroma and epithelium (in white) and between stroma and endothelium (in yellow). Cell nuclei were stained with DAPI. Scale bar represents 50 μm.

In the limbal/corneal epithelial branch, clusters 1–4 were identified as limbal cell states based on the expression of stem cell and limbal marker genes. Cluster 1 and cluster 2 both expressed limbal markers *CXCL14* (26) and *KRT14* (27) and the stem cell marker *TP63* and were therefore annotated as LSCs (Fig. 1B). PROGENy pathway analysis (28) of HVGs revealed WNT pathway enrichment in both clusters (Fig. S3). Among these two clusters, cluster 1 (LSC-1) highly expressed *KRT15* (29) and *GPHA2*. *GPHA2* has been associated with early/quiescent LSC state (30). In cluster 1, we also identified a novel marker gene *SLC6A6*. Cluster 2 (LSC-2) expressed *CPVL* that was reported as an LSC niche marker (18). We confirmed marker gene expression at the protein level by immunochemical staining. SLC6A6 and CPVL were both expressed in the limbal region and showed small overlap with p63 (Fig. 2A and B). LSC-2 also expressed differentiation-related genes like *KRT3* at a low level, suggesting that LSC-2 represents an early stage of differentiated LSCs (31). Clusters 3 and 4 both expressed the epithelial marker *KRT14* and *AREG*, sharing Gene Ontology (GO)-term (32) enrichment for epidermal development (Fig. 1C) and the EGFR pathway (Fig. S3), but had low *TP63*, *KRT15, LAMA5*, and *ITGB4* expression. They were annotated as LSE. These two clusters differed slightly in the marker gene expression, *CXCL14* and *GJB6* higher in cluster 3, and *S100A2* and *AREG* higher in cluster 4.

Other clusters (5–9) in the limbal/corneal epithelial branch all expressed the epithelial mucosal barrier marker *CXCL17* (33) (Fig. 1B), and HVGs were strongly enriched for the epidermis development (GO) (Fig. 1C) and for the p53 pathway (Fig. S3). Clusters 5 and 6 were annotated as Cj cells, marked by the expression of *AQP5* (34), *MUC1* (35), *KRT7* (36), *KRT13* (18), and *S100A9* (37). Pathway analysis of these Cj clusters showed their strong link to immune responses, such as neutrophil degranulation, neutrophil activation, and the TNF-α pathway (38) (Fig. S3). Clusters 7–9 were annotated as central epithelium (CE) cells, as they all expressed *KRT3* and *KRT12* (31). Cluster 7 had high *LAMA5* and *ITGB4* expression, typical for basal cells, and low *AREG* and *KRT24* expression and was therefore annotated as basal CE. In contrast, cluster 9 had low *LAMA5* and *ITGB4* expression, but higher *KRT24* (39) and *LYPD2* (17) expression, typical for superficial CE cells. Cells from cluster 8 showed an intermediate expression pattern, indicating that this cluster contained wing CE cells.

In the nonepithelial branch, clusters 10–15 were identified as stromal cells due to their expression of the corneal stroma marker *LUM* (40) (Fig. 1B). The clusters separated into multiple distinct cell states and were annotated as SK and fibroblast clusters. Among these clusters, cluster 10 was identified as qSKs due to its high expression of *LUM* and *KERA* (40) as well as qSK markers *CD34* and *AQP1* (41). Notably, cells from this cluster expressed high levels of the TF *POU3F3*, a novel marker gene for qSK. By costaining stromal cells with the keratocyte marker keratocan (encoded by *KERA*) (Fig. 2C), we confirmed that POU domain, class 3 (POU3F3 protein, encoded by *POU3F3)* expression was localized in the nuclei of a small subset of stromal cells (Fig. 2D), consistent with its mRNA-level expression detected by scRNA-seq in qSK. In contrast to the nuclear expression of *POU3F3* at both mRNA and protein levels in stromal cells, POU3F3 protein was also detected in the cytoplasm of central corneal basal cells where no *POU3F3* mRNA was detected. Clusters 11 and 12 were annotated as SKs as they displayed lower *CD34* expression but high levels of the SK marker *KERA* (40). Cluster 13 cells expressed *MMP1* and *MMP3*, known for their involvement in SK extracellular matrix remodeling and mechanical stress responses (42), which are critical processes in SKs that transition into a repair-like phenotype (43). We therefore annotated this cluster as transitional stromal keratocytes (TSK). Clusters 14 and 15 were defined as CFs because cells in these clusters exhibited high expression of general fibroblast markers *FBLN1*, *COL1A1*, and *COL5A1* (44) (Fig. 1B). We confirmed the expression of the fibroblast marker fibulin 1 (encoded by *FBLN1*) in a subset of stromal cells, likely to be fibroblasts (Fig. 2E). GO and pathway analyses showed that qSK and fibroblast clusters (10, 14, 15) were enriched in genes involved in extracellular matrix and structure organization (Fig. 1C), and in androgen, estrogen, TNF-α, and PI3K signaling pathways (Fig. S3), known to be involved in stroma cell function (45).

Clusters 16–21 within the nonepithelial branch displayed nonstromal identities and had a relatively small number of cells. Cluster 16 was identified as CEC due to its unique expression of *CA3*, *COL8A2*, and *SLC4A11* (46) (Fig. 1B). Interestingly, cells in this corneal endothelial cluster expressed high levels of *TNNC1*, which was validated by protein staining of troponin C1 (Fig. 2F). Although the function of troponin C1 in CEC is not yet clear, *TNNC1* may be a novel marker for this cell state. Furthermore, cluster 16 exhibited enrichment in GO terms related to ATP metabolism, oxygen-level regulation (Fig. 1C), and the hypoxia pathway (Fig. S3), consistent with the known function of CEC in fluid pumping that requires energy (47). Cluster 17 was annotated as blood and lymph endothelial cells since most cells uniquely expressed the blood vessel marker *ACKR1* (Fig. 1B). Additionally, this cluster expressed the lymph vessel marker *LYVE1* (48) and displayed enrichment in the vascular endothelial growth factor pathway (Fig. S3). Cluster 18 was annotated as melanocytes (Mel) due to its high expression of the melanocyte markers *PMEL*, *MLANA*, and *TYRP1* (49) (Fig. 1B). Cluster 19 was identified as IC because its cells specifically expressed *CCL3* and *CCL4*, markers for T cells and macrophages (50) (Fig. 1B), respectively, and demonstrated significant enrichment in functions such as T cell activation, positive regulation of cytokine production (Fig. 1C), and the NF-κB pathway (Fig. S3). The smallest cluster 20 was annotated as nm-cSCs as its cells exclusively expressed Schwann cell markers such as *SOX10, CDH19*, *NGFR,* and the nm-cSC marker *SCN7A* (51) (Fig. 1B). Lastly, cluster 21, a cluster previously defined as fibroblast CEC by Collin et al. (17) (Figs. S1C and S2), was annotated as mural cells (MC) since cells of this cluster expressed unique MC markers *ACTA2*, *NOTCH3*, and *MYL9* (44) (Fig. 1B).

In summary, we created a corneal cell state meta-atlas that contains more comprehensive annotations of corneal cell states and their associated marker genes, through integrating multiple scRNA-seq datasets.

### Developing a machine learning–based prediction tool for human corneal cell states using the meta-atlas as input

To facilitate corneal cell state analysis in future scRNA-seq studies, we constructed cPredictor, a machine learning pipeline that leveraged cell state annotations of our meta-atlas to train an SVM model (Fig. S5A). An SVM was selected since this model works well for scRNA-seq dataset annotations (52), and it enables straightforward model explainability. To do this, we first performed four rounds of recursive feature elimination using SHapley Additive exPlanations (SHAP (53)), reducing the feature space to 1,243 genes (consisting of 1,047 HVGs and 196 non-HVGs) that retained known marker genes of corneal cell states, such as *PAX6, KERA, FLBN1, SCL4A11, SCN7A,* and *NOTCH3* (Fig. 1B). We then conducted hyperparameter tuning on the regularization parameter, class weights, and number of iterations (Materials and methods) and performed a 5-fold cross-validation with the 1,243 selected genes. This resulted in our final model hyperparameters: 0.01 for the regularization parameter, balanced class weights, and 1,000 maximum number of iterations, with a model performance of a weighted F1 score of 95.75% across all classes (Fig. S5B). Model calibration was performed to ensure that the model-predicted cell state certainty scores closely resembled the observed probabilities during model training. In addition to cell state prediction, cPredictor also outputs common machine learning scores and calibration plots, indicating both the performance of the trained model and calibration for each class. Moreover, cPredictor generates pretrained models from which the top *n* genes driving the cell state predictions (top explainable genes) can be investigated by explainable AI methods, such as SHAP.

**Fig. 3.**
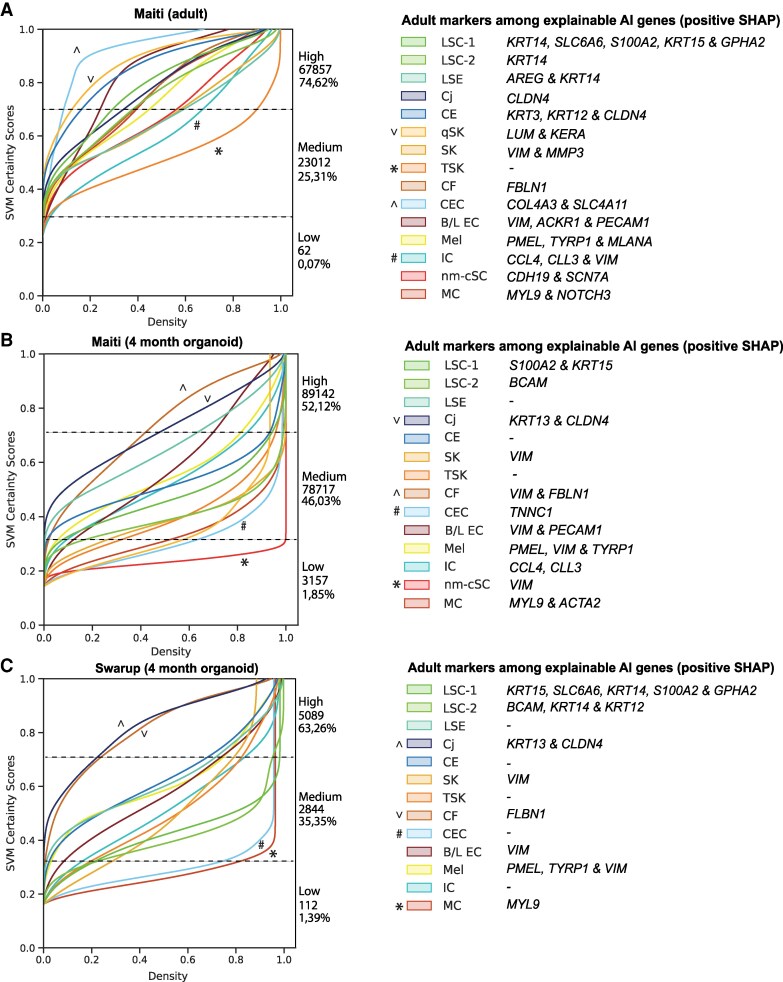
A machine learning–based prediction pipeline, cPredictor, for human corneal cell states in scRNA-seq datasets. Prediction certainty plots of corneal cell states on adult corneal cells (A), 4-month-old organoids from Maiti (54) (B), and 4-month-old organoids from Swarup (55) (C). Left, the *x*-axis shows the cumulative kernel densities and the *y*-axis depicts the model confidence (SVM certainty score). The numbers and percentages of cells corresponding to low (<0.3), medium (>0.3 and <0.7), and high (>0.7) certainty scores in each dataset are depicted next to the plots. ^/V indicates cell states most similar, and */# indicates cell states least similar to corneal cell states from the meta-atlas. Right, corneal markers among the top 10 explainable AI genes driving model's decisions for each of the predicted corneal cell states determined by their positive SHAP values are shown.

To test the performance of cPredictor, we applied it to predict the corneal cell states on one extra dataset from human adult corneas (54) that was not included in model training and cross-validation. We expected that, if cPredictor, trained on the human cornea data, performs well, it should be able to annotate cell states with high confidence scores in this dataset. As expected, cPredictor annotated cell states with high confidence (certainty scores >0.7 on a scale of 0–1) for most cells (∼75%; Fig. 3A). The remaining cells (∼25%) had medium certainty scores (>0.3 and <0.7), and only 62 cells (<0.1%) showed low certainty scores (<0.3). Compared with that original study (54) in which 12 clusters were identified, cPredictor identified all 15 clusters with high or medium certainty scores. Among these clusters, CEC and qSKs had the highest certainty scores (>0.7) and IC and transitioning stromal keratocytes (TSK) had the lowest scores (<0.3) compared with all other cell states. Different limbal and corneal epithelial cell states showed high and medium certainty scores, accompanied with known marker genes (Fig. 1B) among the top 10 explainable genes (Fig. S6A). These included *KRT14*, *SLC6A6* and *S100A2, KRT15*, and *GPHA2* for LSC-1, *KRT14* for LSC-2, *AREG* and *KRT14* for LSE, *KRT3*, *KRT12,* and *CLDN4* for CE, and CLDN4 for Cj. Cells from nonepithelial cell states also showed high and medium certainty scores with known markers among the top explainable genes, such as *LUM* and *KERA* for qSK, *VIM* and *MMP3* for SK, *FBLN1* for CF, *COL4A3* and *SLC4A11* for CEC, *VIM*, *ACKR1*, and *PECAM1* for B\L EC, *PMEL*, *TYRP1*, and *MLANA* for Mel, *CCL3*, *CCL4*, and *VIM* for IC, *MYL9* and *NOTCH3* for MC, and *CDH19* and *SCN7A* for nm-cSC. It is worth noting that, in this dataset, cPredictor also annotated 196 cells as MC (0,2%) and 101 cells (0,1%) as nm-cSCs that were not identified in the original study, likely due to their small number.

To summarize, we demonstrated that cPredictor was able to predict cell states including the rare types on a scRNA-seq dataset of the human cornea.

### Assessing cell states in iPSC-derived corneal organoids using cPredictor

Corneal organoids derived from iPSCs represent good models to study corneal biology and pathogenesis. However, how cell states in iPSC-derived corneal organoids resemble those in human cornea is still an open question. To address this, we collected two studies where scRNA-seq was performed on iPSC-derived corneal organoids: one from Maiti et al. (54) using 4-month-old organoids and the other from Swarup et al. (55) containing time-series data from 1-month-old organoids up until 4-month-old organoids.

First, we applied cPredictor to the two scRNA-seq datasets of 4-month-old organoids (54, 55) to determine cell states, as the 4-month-old organoids may be most similar to the corneal cell state meta-atlas that was created using data from human adult corneas. In the study of Maiti et al. (54), cPredictor annotated ∼52% of the cells with high certainty scores (>0.7) and ∼46% with medium certainty scores (>0.3 and <0.7). There were a small number of cells annotated with low certainty scores (∼2%; Fig. 3B). Limbal/corneal–epithelial cell states mainly showed medium certainty scores. Compared with cell states in the meta-atlas (Fig. 3A), there was a reduction in the number of markers among the top explainable genes (Figs. 3B and S6B). These included *SLC6A6* and *KRT15 for LSC-1*, *BCAM* for LSC-2, and no well-known markers for LSE and CE. Cj showed the highest certainty scores (>0.7) among epithelial cell states, together with *KRT13* and *CLDN4* among the top explainable genes. Most nonepithelial cells showed medium certainty scores, with a reduced number of markers among the top explainable genes (Figs. 3B and S6B). This included *VIM* for the majority of cell states, *TNNC1* specifically for CEC, *PMEL* and *TYRP1* for melanocytes (Mel), *CCL3* and *CLL4* for IC, and *MYL9* and *ACTA2* for MCs. Among them, CF showed high certainty scores (>0.7) together with *VIM* and *FBLN1* among the top explainable genes. Moreover, nm-sSC showed low certainty scores (<0.3) without *CDH19* and *SCN7A* among the top explainable genes, and no cells were predicted as qSKs.

In the other study where 4-month-old organoids were also generated (55), cPredictor showed similar annotation of cell states, ∼63% showed high, ∼35% showed medium, and ∼1% showed low certainty scores (Fig. 3C). Similar to the data from Maiti et al. (54), Cj and CF cells showed the highest certainty scores (>0.7), contributing to the majority of cells having the highest certainty scores, and none of the cells were predicted as qSK. LSC-1 and LSC-2 also showed similar certainty scores, as compared to those from Maiti et al., but with additional markers in the top explainable genes, *KRT14* and *GPHA2* for LSC-1 and *KRT12* and *KRT14* for LSC-2. Other differences included MCs, which showed very low certainty scores (<0.3), with only *MYL9* among the top explainable genes, and no nm-cSC cells were predicted. As expected, cell states from earlier timepoints of organoids (months 1–3) showed even less cells having high certainty scores (>0.7): 29% at 1 month, 36% at 2 months, and 37% at 3 months (Fig. S5C–E), with a limited number of well-known markers among the top explainable genes (Fig. S6D–F).

Taken together, our cPredictor results showed a large difference between cell states of iPSC-derived corneal organoids and those of the human adult corneas, suggesting more immature cell states in corneal organoids.

### Integrating human cornea scATAC-seq data with the corneal cell state meta-atlas

Having obtained comprehensive cell states in the human cornea, we sought to identify key regulators that govern cell state determination as the second application of the human corneal cell state meta-atlas. For this, we used scATAC-seq data of the human cornea (17). To combine this dataset with the scRNA-seq-based corneal cell state meta-atlas, we employed the Seurat label transfer method to label the cell state of each single cell in the scATAC-seq data. This method matches DNA accessibility signals at genomic regions near expressed genes detected in scRNA-seq and predicts and labels scATAC-seq cells according to the cell states in scRNA-seq. In addition, the confidence of the prediction is represented by a model prediction score. Using this method, we successfully identified all cell states that were defined in the meta-atlas in the scATAC-seq, except MC (Fig. S4A). Since scATAC-seq data were known to be sparse, we selected cells with a model prediction score of 0.4 or higher (range 0–1) and clusters consisting of at least 100 cells as reliable data for downstream analyses. This gave rise to cell clusters LSC-1, LSC-2, LSE, CE, Cj, qSK, and CF (Fig. S4B). Among limbal/corneal epithelial cell states, LSC-1 appeared to be clearly distinct from others, whereas other limbal/corneal epithelial cells (LSC-2 and LSE, CE, Cj) clustered together, based on scATAC-seq. Predicted Cj cells had the highest contribution among cell states in scATAC-seq. qSK and CF were distinct from the limbal/corneal epithelial cells and separated into small clusters (Fig. S4B).

To confirm the predicted cell states in scATAC-seq through label transfer, we examined scATAC-seq signals near a select subset of marker genes from scRNA-seq (Fig. 1B). Among limbal/corneal epithelial cell states, the scATAC-seq signal of the marker gene *KRT15* was highest in LSC-1, consistent with its expression in scRNA-seq (Fig. 1B), indicating appropriate label transfer prediction for this cell state (Fig. S4C). *LYPD2*, an identified marker gene for CE, displayed a high scATAC-seq signal in CE (Fig. S4C). As expected, Cj cells displayed the highest accessibility for *S100A9*, a Cj marker gene (Fig. S4C). In the stromal cell states, the highest accessibility of the marker gene *KERA* was observed in qSK, as compared to other cell states (Fig. S4C). Similarly, the fibroblast marker *FBLN1* exhibited high accessibility in CF (Fig. S4C). These results indicated that the label transfer method gave reasonably accurate prediction of cell states using scATAC-seq data, but also showed less distinct separation of cell states, as compared to using scRNA-seq.

### Prediction of key TF using motif analysis on accessible chromatin regions

Next, to predict binding of key TFs driving cell states, we generated pseudobulk for each cell state by merging cells from cell states and subsequently performed TF motif enrichment analysis on accessible chromatin regions detected by scATAC-seq. We then compared the TF motif enrichment scores with pseudobulk gene expression of linked TFs through correlation analysis. This identified expressed TFs binding to accessible chromatin regions and potentially highly important in driving gene expression in specific cell states.

**Fig. 4.**
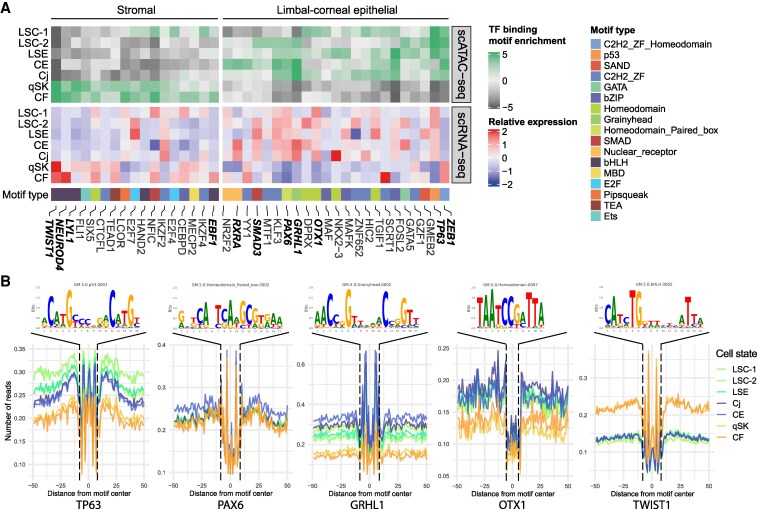
Motif enrichment and prediction of TF binding in cell states of the human cornea: A) heatmaps of the motif scores of the top 10 enriched TFs (upper panel) and of TF expression levels (lower panel) for each cell state. The bottom panel shows the type of associated motif. B) Examples of the consensus motif and TF footprints from 5A. Names of cell states: LSC-1, limbal stem cells 1; LSC-2, limbal stem cells 2; LSE, limbal suprabasal epithelium; CE, central epithelium; Cj, conjunctiva; qSK, quiescent stromal keratocytes; CF, corneal fibroblasts.

We identified two distinct groups of TFs associated with either the limbal/corneal epithelial or the nonepithelial branch. The group associated with the limbal/corneal epithelial cell states included many known TFs in corneal LSCs or epithelial cells such as TP63, FOSL2, PAX6, GRHL1, OTX1, SMAD3, and RXRA (Fig. 4A). Among these TFs, TP63 showed most specific high scATAC-seq signals at the TP63 motif in all three limbal cell states, namely LSC-1, LSC-2, and LSE (Fig. 4A and B), consistent with the highest expression in LSC-1. SMAD3 displayed a high TF-binding enrichment score only in LSC-2, despite its broader expression (Fig. 4A). FOSL2, PAX6, and GRHL1 showed high TF-binding motif enrichment scores in several limbal and epithelial cell states (Fig. 4B), consistent with their relatively high gene expression in these cell states (Fig. 4A and B). OTX1 had high TF-binding motif enrichment scores in both LSC-1 and Cj, in line with its gene expression in these cell states. RXRA that was highly expressed in CE showed high TF-binding enrichment score unique to CE (Fig. 4A). Interestingly, ZEB1 exhibited an inverse relationship between TF-binding motif enrichment and gene expression, suggesting a repressor role of this TF in limbal/corneal epithelial cells.

The group of TFs that had high TF-binding enrichment scores in cell states of the nonepithelial branch included TWIST1, NEUROD4, LYL1, and EBF1 (Fig. 4A), all of which are TFs associated with bHLH motifs. As expected, the region surrounding the TWIST1-associated motif consistently displayed high scATAC-seq signals in both qSK and CF (Fig. 4E).

### Prediction of key TFs using GRN analysis

**Fig. 5.**
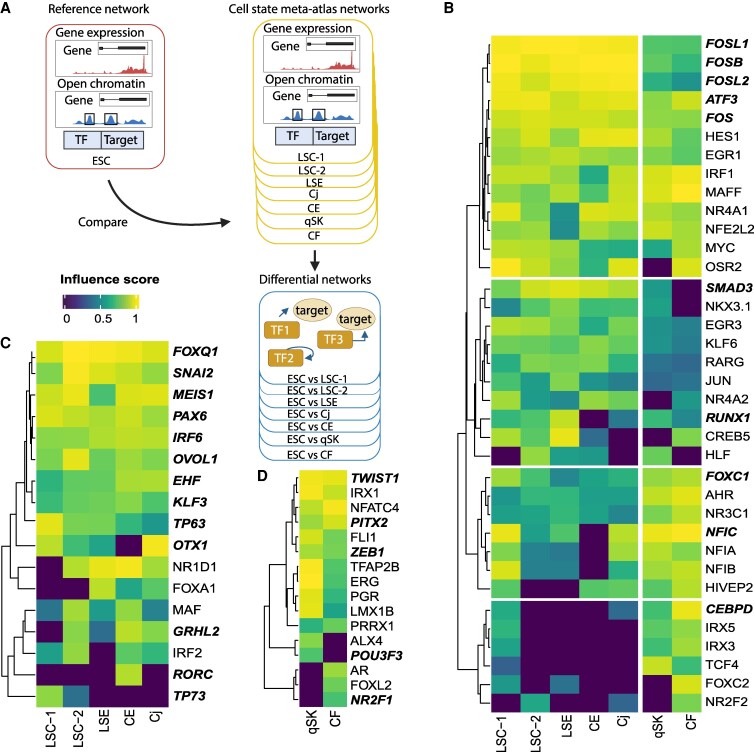
Prediction of key TFs controlling corneal cell states using GRN analysis of scANANSE. A) An in vitro single-cell human embryonic stem cell (hESC) network was used as a reference against networks of all individual cell states in the corneal cell state meta-atlas. Black boxes enclosing peaks in open chromatin depict putative enhancers where TFs can bind. B) Heatmap of influence scores (>0.8) of TFs shared between limbal/corneal epithelial and stromal cell states, predicted by scANANSE. C) Heatmap of influence scores (>0.8) of TFs specific for limbal/corneal epithelial cell states, predicted by scANANSE. D) Heatmap of influence scores of the top 40 TFs specific for stromal cell states, predicted by scANANSE. Names of cell states: LSC-1, limbal stem cells 1; LSC-2, limbal stem cells 2; LSE, limbal suprabasal epithelium; CE, central epithelium; Cj, conjunctiva; qSK, quiescent stromal keratocytes; CF, corneal fibroblasts.

Using motif analysis on accessible chromatin regions predicts TFs that regulate gene expression in specific cell states, but it does not indicate the importance of TFs in driving cell state determination. To predict the importance of TFs driving corneal cell state identity, we leveraged our previously developed single-cell GRN method, scANANSE (16), which ranks the importance of a TF represented by an influence score in a specific cell state, as compared to another. For this comparison, embryonic stem cells (56) were used against all cell states in the human corneal cell state meta-atlas (Fig. 5A), a strategy that was previously shown to be effective for TF identification in similar cell states when applying the ANANSE pipeline (57). In this analysis, we identified TFs with high influence scores shared in all cells (Fig. 5B) but also those distinct to each branch (Fig. 5C and D).

Among the shared TFs, we identified four groups (Fig. 5B). The first group included FOSB, FOSL1, FOSL2, FOS, and ATF3, TFs associated with the AP-1 complex. They had high influence scores in both epithelial and nonepithelial branches, with the scores in the epithelial branch slightly higher than those in the nonepithelial branch. Among these five TFs important for regulating cell states in the human cornea, only FOSL2 was detected in our motif analysis (Fig. 4A). The second group contained TFs with high influence scores in epithelial cell states and lower, but detectable scores in qSK and CF. This group contained SMAD3 and RUNX1, with a known function associated with LSCs (14). The third group contained TFs such as FOXC1 (14) and NFIC that had high influence scores in both branches but with higher scores in the nonepithelial branch; one of these, FOXC1, was not identified using motif enrichment analysis. The fourth group of TFs had high influence scores in cell states not specific to a branch. Remarkably, this group contained CEBPD that had high influence scores in LSC-1 as well as in qSK and CF, even though this TF was known to be associated specifically with LSCs (58).

Many identified TFs with high influence scores specific for limbal/corneal epithelial cell states are well-known epithelial TFs. These included EHF, KLF3, TP63, and PAX6 (Fig. 5B), which are known for their role in corneal differentiation (59), and FOXQ1, SNAI2, MEIS1, and OVOL1, which are known to be involved in WNT signaling (60–62). Other TFs with high influence scores were OTX1, GRHL2, RORC, and TP73. OTX1 exhibited the highest influence scores in LSC-1 and Cj, which was consistent with our motif analysis (Fig. 4A). GRHL2 showed a high influence score for LSC-2, CE, and Cj. RORC showed a unique high influence score in CE, while TP73 had a high influence score in LSC-1 and a lower influence score in LSC-2.

Many of the identified nonepithelial specific TFs are less known in the cornea. Most of them, including PITX2, LMX1B, TWIST1, FLI1, ERG, and ZEB1, exhibited high influence scores in both qSK and CF (Fig. 5D), with TWIST1, FLI1, ERG, and ZEB1 being consistent with motif analysis (Fig. 4A). ALX4 and POU3F3 both had high influence scores only in qSK (Fig. 5D), in line with their roles associated with maintaining mesenchymal identity (63). To note, POU3F3 is one of the novel markers identified in this study, and its expression at the protein level was validated (Figs. 2B and 3E). In contrast, NR2F1 displayed a high score only in CF (Fig. 5D).

Taken together, our analysis identified well-known and novel key TFs for corneal cell state determination. We also showed that corneal cell states are mostly driven by combinations of TFs, whereas a small number of TFs were cell state specific. scANANSE's influence scores of TFs and general gene expression across these cell states can be interactively explored in a dashboard (https://huggingface.co/spaces/Zhou-group/corneal_cell_state_meta_atlas).

## Discussion

Understanding the precise cell states is pivotal for both in vivo and in vitro studies on development, pathogenesis, and regeneration of the cornea. In this work, by integrating four scRNA-seq datasets, we annotated corneal cell states including previously unknown rare cell states, identified novel marker genes, and created a corneal cell state meta-atlas. We demonstrated that the machine learning–based prediction pipeline cPredictor that applies the cell state meta-atlas as the reference can define cell states in various types of corneal scRNA-seq data. Furthermore, we characterized distinct combinations of key TFs controlling cell states of the human cornea, by integrating scATAC-seq with our scRNA-seq-based cell state meta-atlas. Both marker gene expression and TF influence scores can be interactively visualized through a web portal (https://huggingface.co/spaces/Zhou-group/corneal_cell_state_meta_atlas). This portal together with cPredictor will evolve continuously as an expanding resource for investigating human corneal cell states.

The corneal cell states characterized in this work are mostly consistent with known corneal cell states in the literature (8, 51, 64, 65). Two populations of LSCs were detected in our study; LSC-1 expressed the quiescence-related gene product *GPHA2*, while LSC-2 showed significant enrichment for genes linked to epidermal differentiation, indicating a more differentiated state of LSC-2 compared to LSC-1. These findings are in line with previous work showing one LSC population exhibiting quiescent stem cell traits and another active in corneal regeneration in mice (65). Interestingly, our integrated corneal cell state meta-atlas supported the existence of a tiny number (<0.15%) of nm-cSCs, which has been reported across various species (51, 64, 66). Although the mechanisms behind nm-cSC function are not fully clear, these cells potentially play important roles in corneal wound healing (66) and in cornea-associated diseases such as familial dysautonomia (67). It is worth noting that the nm-cSC cell state was not identified in any of the individual scRNA-seq studies, likely due to its small cell number in the cornea.

We uncovered novel marker genes linked to corneal cell state identities in the integrated scRNA-seq cell state meta-atlas. Of note, we detected *SLC6A6* as a novel marker specific for the LSC state LSC-1, with high protein abundance limited to the corneal limbus. The presence of *SLC6A6* in the limbus is in line with a recent single-nucleus RNA-seq study (68). *SLC6A6* plays roles in reducing reactive oxygen species (ROS) and in regulating the Wnt/β-catenin signaling pathway (69), a signaling pathway known to be important in the limbus. In LSC-1, *SLC6A6* might be an important player in the Wnt/β-catenin pathway. In this work, several WNT-associated TFs FOXQ1, SNAI2, MEIS1, and OVOL1 were identified in our GRN analysis and showed high influence scores in LSC-1. Further investigation of this Wnt/β-catenin axis is warranted, as none of the WNT-associated TFs have been previously implicated in the regulation of LSCs. Furthermore, *TNNC1*, a well-known cardiac cytoskeletal troponin gene (70), was identified as a distinct marker gene for the corneal endothelium and validated in our immunohistochemical staining of human corneas. Its potential role as a calcium sensor (70) possibly needed for endothelial cell function in the human cornea needs to be further investigated.

The strength of the corneal cell state meta-atlas and its derived SVM-based machine learning pipeline cPredictor in the identification of rare cell states was demonstrated in our study. So far, only one scRNA-seq study of the human cornea (54) was available for us to test its performance. cPredictor was able to predict 15 cell states with medium to high confidence scores, whereas only 12 cell states were predicted in the original study (54). Furthermore, our corneal meta-atlas seems to be more robust as a corneal reference, as it was integrated from multiple datasets derived from different methods of cell retrieval (13), and therefore contained more cell states than each individual study. We showed that the data from Collin et al. (17) contributed to the majority of epithelial cells in the cell state meta-atlas. Additionally, this was the only study to retrieve MCs, previously annotated as fibroblast CEC, probably due to tissue handling as they used bulk enzymatic sample disaggregation. In the study of Català et al. (18), a dissection protocol that gently separates the cornea in multiple parts before disaggregation was carried out to retrieve high-quality corneal stromal cells, including SKs. Therefore, a small number of (transitioning) SKs were only retrieved in this study (18). Nevertheless, our constructed corneal cell state meta-atlas together with cPredictor should be more robust to predict corneal cell states from different retrieval methods.

With its automated capabilities, cPredictor is easy to use. Our containerized software and command-line-based approaches enable ease-of-use for predicting adult corneal cell states in external datasets. It is also straightforward to incorporate new datasets in cPredictor, if new scRNA-seq datasets of the human cornea are available. This may further strengthen its capability to detect rare cell states and marker genes. Additionally, cPredictor could potentially incorporate other meta-atlases as training datasets, e.g. from mouse cornea data or other tissues, expanding its applicability beyond human cornea research. Moreover, our approach to data integration and its direct applications could be used both for constructing meta-atlases for other tissues and for other model organisms.

Furthermore, applying cPredictor allowed us to investigate cell states in iPSC-derived corneal organoids, using the corneal cell state meta-atlas as the reference. As the meta-atlas was generated from four scRNA-seq datasets derived from adult human corneas (17–20), cell states in organoids were compared to those in human adult corneas. Our results confirmed that current iPSC-derived corneal organoids do not yet resemble the cell states in adult corneas. Our predictions showed that Cj and CF cell states in 4-month-old corneal organoids were most similar to the adult cell states, represented by certainty scores and detected marker genes. This suggests that studying Cj and CF using organoids could give relevant information on their cell functions in the cornea. However, many cell states, like CEC, still show large differences, as compared to those in the adult cornea. The clear differences in the cell states between the adult human corneas and iPSC-derived corneal organoids suggest that corneal organoid generation needs further improvement, to be able to fully mimic the function of the cornea, potentially via maturation. That maturation may be one of the bottlenecks is supported by the observations that organoids generated at the end of months 1–3 were less similar to the reference, as compared to the 4-month-old organoids. Prolonging the culturing time of the organoids could be one strategy. Alternatively, proper environmental cues, such as signaling molecules or mechanical force, could improve the maturity or functionality of cells in corneal organoids. Such information may be derived from further in-depth studies on the similarities and differences between the human adult corneas and iPSC-derived corneal organoids.

The identified TFs through scANANSE GRN analysis revealed many well-known key (corneal) epithelial TFs, including PAX6, TP63, SMAD3 (14), GRHL2, and FOSL2 (57), in all limbal/corneal epithelial cell states. TFs in nonepithelial cell states qSK and CF included PITX2, POU3F3, ALX4, LMX1B, TWIST1, FLI1, and ERG. Except PITX2 that is known to play important roles in neural crest specification and proper development of the corneal stroma (71), other TFs are less studied in the cornea. POU3F3, of which the protein was localized in the cell nuclei of the corneal stroma, was identified as a novel TF and marker gene for qSK in our study. However, protein expression of POU3F3 was also detected in the cytoplasm in the corneal epithelium, where POU3F3 is probably not functioning as a TF. LMX1B is known for its function in periocular mesenchyme-derived cell identity (72), similar to PITX2, but unknown in the cornea. In addition, the roles of TWIST1, FLI1, and ERG in corneal stromal cell states are completely unexplored. Further research on the function of these TFs is warranted.

In summary, we show that the corneal cell state meta-atlas can serve as a reliable reference for annotating and predicting corneal cell states, e.g. in dissecting the difference between healthy and diseased cells. The easy-to-use computational pipeline cPredictor and the identified marker genes and key TF in various cell states of the human cornea provide a rich resource for follow-up research on corneal biology and regeneration.

## Materials and methods

Additional materials and methods are detailed in Supplemental Information.

### Ethical statement

In this study, two human donor corneas deemed unsuitable for transplantation were used for immunofluorescence analysis. The donors were deidentified with no names or direct identifiers, but the information on sex, age, and clinical history was provided. The corneal tissues were obtained from two male donors, aged 70 and 71 years, from the ETB-BISLIFE Multi-Tissue Center (Beverwijk, The Netherlands). These tissues were preserved in organ culture media at 31 °C. The composition of the media included the following: minimum essential medium supplemented with 20 mM HEPES, 26 mM sodium bicarbonate, 2% (v/v) newborn calf serum (Thermo Fisher Scientific), 10 IU/mL penicillin, 0.1 mg/mL streptomycin, and 0.25 μg/mL amphotericin. Both donor tissues had no history of ocular disease or infection, including HIV or hepatitis B.

### Immunofluorescence

Corneas were halved transversely, embedded in paraffin, and fixed in 4% paraformaldehyde at room temperature (RT) for 10 min. To preserve tissue morphology, 10-μm-thick sections were cut consecutively on adhesive cryofilm type 3C (16UF) using a modified Kawamoto method with slight adjustments (73). For antigen retrieval, tissue sections were incubated in boiling citrate buffer with 0.05% Tween 20 at 95 °C (pH 6) for 20 min or treated with pepsin C (cat. no. ab64201; Abcam) for 10 min at RT. Sections were then permeabilized with PBS containing 0.2% Triton X-100 (cat. no. T8787; Sigma-Aldrich) for 10 min at RT. Blocking was performed in PBS containing 10% goat serum and 2% bovine serum albumin (PBS–2% BSA) for 1.5 h at RT, followed by incubation with primary antibodies (Table S2) diluted in PBS–2% BSA overnight at 4 °C in a humidified chamber. The following day, sections were washed three times with PBS-T and incubated with fluorescently conjugated secondary antibodies (goat antirabbit A488 and goat antimouse A647, both at 1:1,000 dilution; Thermo Fisher Scientific) for 1 h at RT in the dark. Sections were then washed again three times with PBS-T and counterstained with DAPI (1:2,000) for 10 min at RT. After a final set of three washes, coverslips were mounted using Fluoromount-G (Thermo Fisher Scientific). Imaging was performed on an automated inverted Nikon Ti-E microscope equipped with a Lumencor Spectra light source, an Andor Zyla 5.5 sCMOS camera, and an MCL NANO Z200-N TI z-stage, using a CFI S Plan Fluor ELWD 40× objective. Image analysis was carried out using NIS-Elements software.

### Declaration of the use of AI in the writing process

During preparation of this work, generative AI models were used to proofread and improve the fluency of the manuscript text. All text was carefully reviewed and edited as needed by the authors, who take full responsibility of all contents of this publication.

## Supplementary Material

pgaf235_Supplementary_Data

## Data Availability

Datasets used in this study are from previous publications and currently publicly available under the Gene Expression Omnibus identifiers GSE155683, GSE186433, GSE153515, GSE147979, GSE178379, GSE218123, and GSE240458 (17–20, 54–56). All these datasets contain scRNA-seq data. Among these datasets, GSE155683 and GSE178379 also contain scATAC-seq data. The full processing workflow and documentation of code used for Python and R are available in the GitHub repository: https://github.com/Arts-of-coding/Cell-States-and-Key-Transcription-Factors-of-the-Human-Cornea-through-Integrated-Single-Cell-Omics. GRCh38 downloaded with Genomepy version 0.10.0 (74) was used for all downstream analyses. An interactive web app was developed to visualize corneal scRNA-seq meta-atlas, gene expression, and influence scores of TFs. The interactive web app is available at: https://huggingface.co/spaces/Zhou-group/corneal_cell_state_meta_atlas. Single-cell objects of the meta-atlas and files used by cPredictor in this study can be downloaded from Zenodo (75): https://doi.org/10.5281/zenodo.7970736. Exploration of pseudobulk scATAC-seq is available using the link below in the UCSC genome browser track hub: https://mbdata.science.ru.nl/jarts/scATAC_Corneal_meta-atlas/ATAC-seq_trackhub.hub.txt.

## Preprints

The preprint of this study was deposited at BIORXIV/2024/629354.
