# Supplementary material for: Prediction of cell states and key transcription factors of the human cornea through integrated single-cell omics analyses
Source: PNAS Nexus. 2025 Jul 29;4(8):pgaf235. doi: 10.1093/pnasnexus/pgaf235 (PMC12363670; doi:10.1093/pnasnexus/pgaf235)
Supplement: pgaf235_Supplementary_Data [file pgaf235_supplementary_data.zip › PNASNEXUS-PNASNEXUS-2025-00162R-file002.pdf]

Study	Number of donor corneas	Sample disaggregation method	Specifically retrieved regions	Number of scRNA-seq cells in this study	Additional data
Collin et al.	6	Bulk enzymatic	Limbal ring (1), central cornea (1), complete corneas (4)	22276	scATAC-seq
Català et al.	8	Separate dissection	Limbal ring (2), complete corneas (6)	15903	-
Gautam et al.	3	Bulk enzymatic	Whole eyes with cornea data included (3)	10248	-
Li et al.	4	Separate dissection	Limbus after removing central cornea and superficial layers (4)	16609	-
