# Supplementary material for: Prediction of cell states and key transcription factors of the human cornea through integrated single-cell omics analyses
Source: PNAS Nexus. 2025 Jul 29;4(8):pgaf235. doi: 10.1093/pnasnexus/pgaf235 (PMC12363670; doi:10.1093/pnasnexus/pgaf235)
Supplement: pgaf235_Supplementary_Data [file pgaf235_supplementary_data.zip › PNASNEXUS-PNASNEXUS-2025-00162R-file003.pdf]

Primary antibodies	Host	Company	Catalogue Number	Dilution
CPVL	Rabbit polyclonal	Thermo-Fisher	PA5-63308	1:100
SLC6A6	Rabbit polyclonal	Fisher Scientific	16500992	1:100
p63 (ΔNp63)	Mouse monoclonal [4A4]	Abcam	ab735	1:100
Keratocan	Rabbit polyclonal	Sigma-Aldrich	HPA039321	1:100
Fibulin-1	Rabbit polyclonal	Fisher Scientific	16620295	1:100
POU3F3	Rabbit polyclonal	Abcam	ab247159	1:100
TNNC1	Mouse polyclonal [4C2]	Abcam	ab10231	1:100
