# Supplementary material for: Prediction of cell states and key transcription factors of the human cornea through integrated single-cell omics analyses
Source: PNAS Nexus. 2025 Jul 29;4(8):pgaf235. doi: 10.1093/pnasnexus/pgaf235 (PMC12363670; doi:10.1093/pnasnexus/pgaf235)
Supplement: pgaf235_Supplementary_Data [file pgaf235_supplementary_data.zip › PNASNEXUS-PNASNEXUS-2025-00162R-s02.pdf]

PNAS nexus

Supplementary Information for

Prediction of Cell States and Key Transcription Factors of the Human Cornea through Integrated Single-Cell Omics Analyses

Julian A. Arts[1], Sofia Fallo[3], Melanie S. Florencio[3], Jos G.A. Smits[1,2], Dulce Lima Cunha[1], Janou A.Y. Roubroeks[1,5], Mor M. Dickman[4,6], Vanessa L.S. LaPointe[3], Rosemary Yu[1] and Huiqing Zhou[1,2*]
[1] Department of Molecular Developmental Biology, Radboud Institute for Molecular Life Sciences (RIMLS), P.O. Box 9101, 6500 HB Nijmegen, the Netherlands
[2] Department of Human Genetics, Radboud University Medical Center, P.O. Box 9101, 6500 HB Nijmegen, the Netherlands
[3] Department of Cell Biology-Inspired Tissue Engineering, MERLN Institute for Technology-Inspired Regenerative Medicine, P.O. Box 616, 6200 MD, Maastricht, The Netherlands.
[4] University Eye Clinic Maastricht, Maastricht University Medical Center+, P.O. Box 5800, 6202 AZ, Maastricht, The Netherlands.
[5] Department of Clinical Genetics, Maastricht University Medical Center +, P.O. Box 5800, 6202 AZ, Maastricht, The Netherlands.
[6] Department of Ophthalmology, University Medical Center Utrecht, P.O. Box L02417, 3584 CX, Utrecht, The Netherlands.
* To whom correspondence may be addressed: Huiqing Zhou.

Email: j.zhou@science.ru.nl; Jo.Zhou@radboudumc.nl.

This PDF file includes:

Supplementary text
Figures S1 to S6
Tables S1 to S2
SI References

Other supplementary materials for this manuscript include the following:

Supplemental Table S3 (Excel file)

Supplementary Information Text

Methods

Pre-processing single-cell RNA-seq and single-cell ATAC-seq

Datasets were downloaded from GEO with Seq2Science (1). The 10X .sra files were split into fastq files with Seq2Science. Next, Snakemake pipelines were developed to pre-process multiple datasets with cellranger. Using these pipelines, Cellranger count was run with Cellranger 7.0.1 (2), with default parameters and with hg38 to retrieve the matrix, barcodes and features files necessary for downstream analysis. For GSE178379 specifically, Cellranger-arc 2.0.2 was run to retrieve these files for multi-omics data. Comparable to scRNA-seq pre-processing, a Snakemake pipeline containing Cellranger-atac count was run with Cellranger-atac 2.0.0 (3) to generate both the position sorted bam file and the singlecell.csv file, containing barcode information, necessary for snapATAC (4) analysis in Bash and R.

Quality control of scRNA-seq and scATAC-seq

scRNA-seq datasets were analyzed in R with Seurat version 4.0.2 (5), as described in the GitHub repository. scRNA-seq cells were selected with a minimum count of 2000, a feature number higher than 1000 and a mitochondrial percentage lower than 30 percent. Expected doublets were removed with DoubletFinder version 2.0.3 (6). scATAC-seq datasets were processed in R with SnapATAC version 2.0[116]. Due to the higher sparsity of the data compared to scRNA-seq, a strict quality control was used. Only cells with a mitochondrial ratio lower than 30 percent were selected. Additionally, the fragment threshold number for each cell was set between 5000 and 500.000. To minimize the number of doublets that could skew the data, a maximum of 500.000 for the fragment and a number between 3.75 and 5 for the unique count were chosen. A Log10 coverage above 3.6 and a promoter ratio between 2 and 8 were used and cells with a duplicate ratio higher than 0.8 and a low mapping quality value higher than 5000 were excluded. scRNA-seq data from GSE218123 and GSE240458 were analyzed in Python with Scanpy version 1.9.2, as described in https://github.com/Arts-of-coding/Cell-States-and-Key-Transcription-Factors-of-the-Human-Cornea-through-Integrated-Single-Cell-Omics. A minimum count of 900 and a mitochondrial percentage lower than 20 percent were used as described by the study of Swarup (7).

Integration and clustering of scRNA-seq datasets

Clustering in each dataset was conducted with specific parameters from dimension numbers (Elbow plot) and with clustering resolution using Clustree version 0.4.4 (8). Clustering on scRNA-seq data from the studies of Català, Collin, Gautam and Li was performed with Leiden clustering (9), using 30, 16, 18 and 24 dimensions and a clustering resolution of 0.15, 0.1, 0.15 and 0.1 respectively. The four datasets were integrated with scVI (scvi-tools version 0.14.0) (10). The parameters for the Variational Autoencoder were selected as 2 for “n_layers”, 30 for “n_latent” and "nb" for “gene_likelihood”. Leiden clustering was performed on the integrated data using a clustering resolution of 0.3. scVI integrated clusters were validated with Harmony integration. Harmony integration was performed with Harmonypy version 0.0.10 (11) with the default parameters as described in the software package.

GO-term enrichment analysis

We performed gene ontology enrichment on all marker genes found in each cell state by use of the org.Hs.eg database (12) for biological processes, with the cut-off of BH-adjusted p-values <0.05 (13). Similarly, GO-terms that had a similarity score regarding overlapping genes of 0.5 (range 0-1) for overlapping genes were joined together using the function simplify from ClusterProfiles version 3.0.4. Further selection on the GO-terms was performed by selecting terms with a minimum gene ratio of 0.1 and terms consisting of at least 200 genes per term. The top 3 highest counted GO terms were visualized.

Pathway enrichment analysis

Pathway enrichment was performed with PROGENy (decoupler-py version 1.6.0), a tool that showed enrichment for 14 curated signal-transduction pathways (14). The top 500 genes for each pathway were selected and no permutations were added. Activity for these curated pathways was inferred for each single cell by running a multivariate linear model from decoupleR. Subsequent visualization was performed in Python.

cPredictor classifier training and prediction with the corneal meta-atlas

All scripts for iterative feature (gene) selection, hyperparameter optimization and model explainability can be found under https://github.com/Arts-of-coding/Cell-States-and-Key-Transcription-Factors-of-the-Human-Cornea-through-Integrated-Single-Cell-Omics. The training dataset supplied to cPredictor (15), using support vector machines from Scikit-learn (Sklearn) (16), was the corneal cell state meta-atlas. Raw scRNA-seq count data was transformed with “log1p” from numpy and MinMax scaling from Sklearn. A 5-fold cross-validation was run on the corneal meta-atlas dataset to estimate generalized model performance. Within the automated pipeline, metrics were logged and calibration for each cell state was generated in a one-versus-all (single-class) fashion. Features (genes) were selected through an iterative approach of gene elimination, starting with expressed genes in the corneal meta-

atlas (Supplementary Information). In each round the top-n highly variable genes and the top-n explainable genes were taken along if the model showed good calibration for that specific cell state. In the end, top explainable genes MALAT1, XIST, SRY, MT1Z, GAS5, BTG2, CD9 and SNHG32 were removed as they contributed to model explanations being either expressed in all cell states or being sex-chromosome specific which negatively affects model predictions. After feature selection, model hyperparameter optimization was performed using Monte-Carlo simulations (17). The hyperparameters in the final model were chosen based both on the optimal ROC-AUC and accuracy scores across classes and can be found under: https://huggingface.co/Arts-of-coding/meta-atlas-cornea-SVM. The Docker container “artsofcoding/cpredictor:v0.4.5_cpredictor_hcornea_v1” was used for cell state predictions on the data from Maiti (18) and Swarup (7).

Recursive feature elimination for cPredictor

In each round, model predictions were explained using their own training data, and the top explainable AI (SHAP) genes were added to the pool of the top highly variable genes (HVG) for each cell state until the model performed well and retained important marker genes for the cornea. For the first round, the top 150 SHAP genes from the approximately 18,000 expressed genes were selected and analyzed using SHAP analysis. In the second round, the top 150 SHAP genes were combined with the top 150 HVGs. In the third round, the top 100 SHAP genes were combined with the top 100 HVGs, and 8 genes were removed (see Materials and Methods). In the fourth round, the top 50 SHAP genes and the top 50 HVGs were selected, but it was observed that relevant marker genes from the third round started missing in this round. In the final round, the top 100 SHAP and HVGs from the third round were used, and hyperparameter tuning was performed.

Prediction of scATAC-seq cells from scRNA-seq data

After annotating the cell clusters in scRNA-seq and performing quality control as described, scATAC-seq cell clusters were imputed with Seurat label transfer with a minimum prediction score of 0.4. The position-sorted bam and singlecell.csv files were used to generate snap files, as described in the SnapATAC package (4).

Bam file generation, transcription factor footprinting and motif analysis

Cell barcodes were retrieved in R to generate a pseudobulk profile of ATAC-signal for each cell state. For downstream analysis, we performed splitting of full bam files by cell states in three of the four datasets, which showed high quality. We performed this barcode splitting for the imputed cell populations consisting of more than 100 cells in scATAC-seq. Bam files were split for cell populations with bamslice based on corresponding cell barcodes with cellranger-dna version 1.1.0 to enable peak calling. Peaks for motif analysis were generated from the .bam files with peak calling software MACS2 (19). Both the bam and Narrowpeak files were used to generate accessible summit files and peak count files with motif analysis software Gimmemotifs (20). The output of Gimmemotifs and Z-score expression of transcription factors were correlated in R to determine which transcription factor was most likely to bind a motif. Footprinting analysis was performed on bam files, bed files and Narrowpeak files of cell states with HINT-ATAC using RGT version 0.13.2 (21). Briefly, rgt-hint footprinting function was used on our Narrowpeak files. Next, the rgt-motif analysis matching function was run on our created bed files. The motifs were matched against the full Gimme Motifs database. Lastly, the footprint files were generated using the rgt-hint differential function. The output files were imported in R to trim the distance from the center of the motif to 50bp upstream as well as 50bp downstream.

Gene regulatory network analysis of single-cell states

scANANSE was performed on single-cell clusters in a pseudo-bulk fashion by using the package AnanseScanpy (22). In short, GRNs of single-cell populations consisting of more than 100 cells present in scATAC-seq were generated. The hg38 genome and the combined pseudo bulk peak matrix, generated with the function export_ATAC_scANANSE of all populations were used for performing ANANSE binding. The output network file from ANANSE binding was used, next to the hg38 as the reference genome for the ANANSE network step. Due to the nature of the 3’ sequencing, CPM values were used as input for generating the network files, generated with the function

export_RNA_scANANSE from the single-cell object. Differential gene expression across single cells was performed by using the export_DEGs_scANANSE function. For ANANSE influence scores, two different networks were compared. A network composed of scATAC-seq and scRNA-seq from human naïve embryonic stem cells (23) (see data availability) was used as a reference for each of our cell states in the corneal meta-atlas. All corneal cell state meta-atlas networks were compared to this network in the scANANSE influence step. These binding, network and influence steps were performed with ANANSNAKE, as described in the scANANSE workflow.

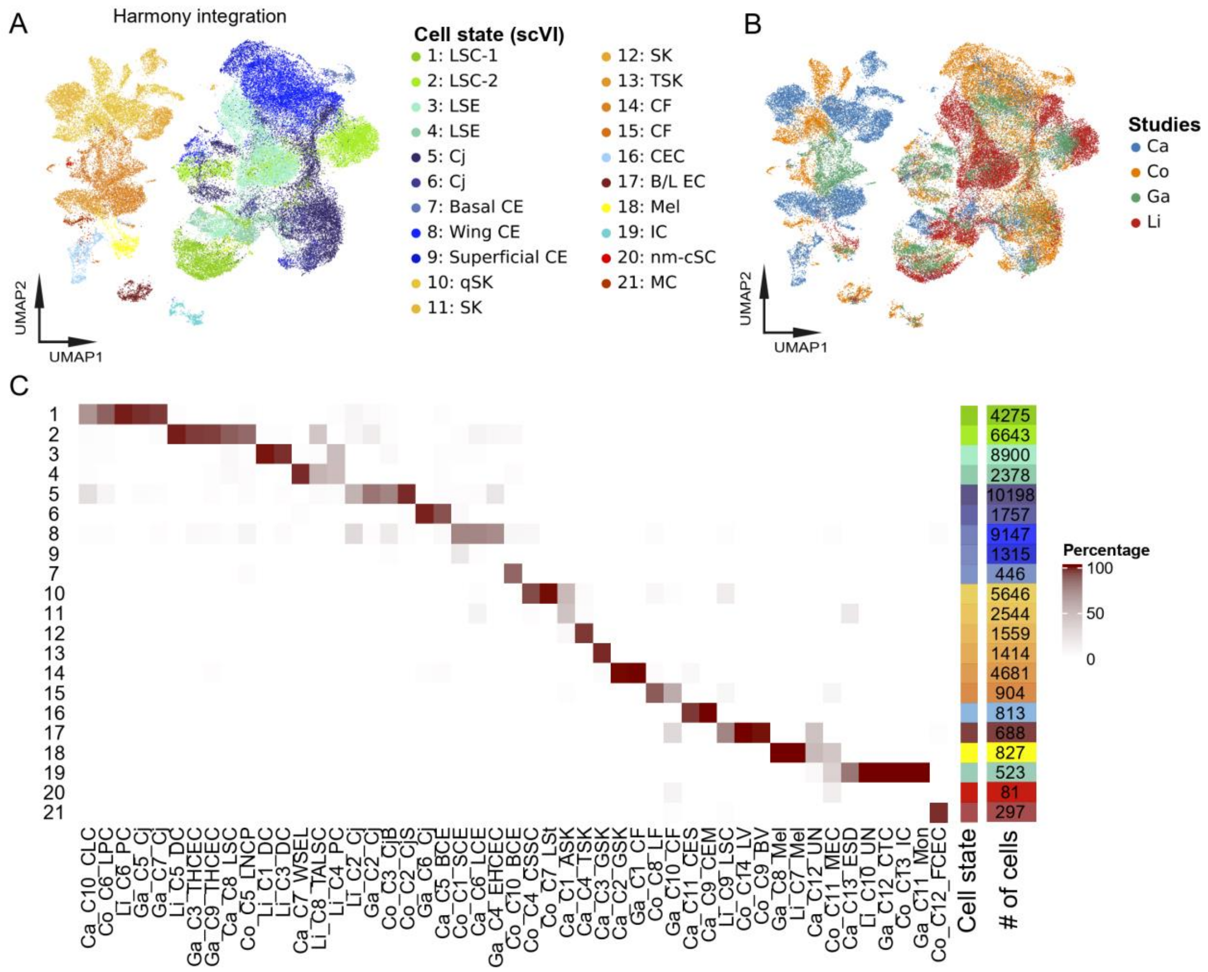

Fig. S1. Data integration with Harmony and distribution of integrated cell clusters with defined cell states across studies. (A) Integration with Harmony with colors representing cell states defined with scVI integration. (B) Harmony integration with colors representing studies. Ca = Català, Co = Collin and Ga = Gautam. (C) Cell numbers and cell states in the integrated data are annotated on the right, and the original cell states in individual studies (figure S2) are indicated on the x-axis. Ca stands for Català, Co stands for Collin, Ga depicts cells from Gautam and Li indicates cells from Li. C indicates the cluster number across individual studies. The cell origins from A are depicted on the right, together with the total number of cells.

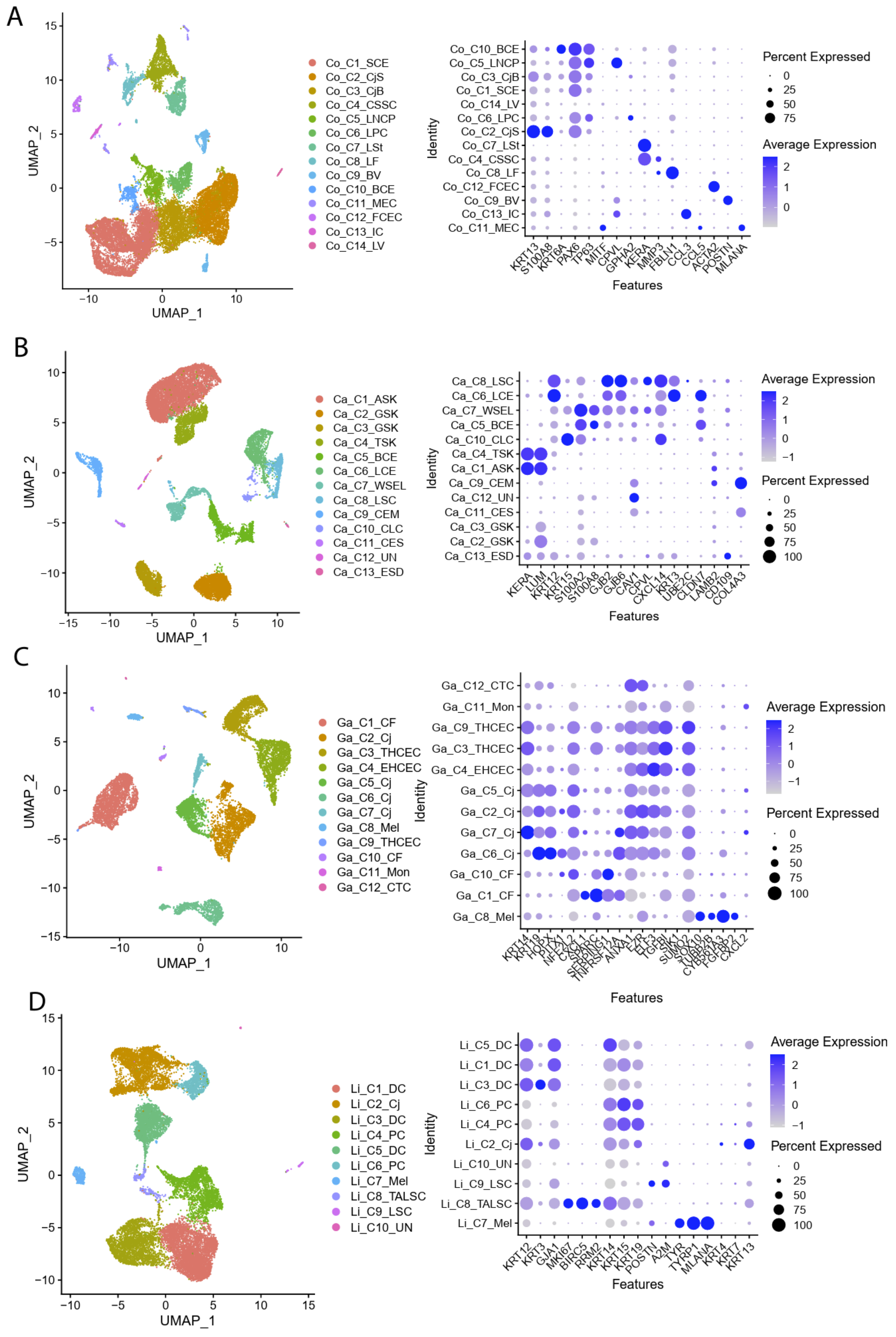

A
UMAP_2
UMAP_1
Co_C1_SCE
Co_C2_CjS
Co_C3_CjB
Co_C4_CSSC
Co_C5_LNCP
Co_C6_LPC
Co_C7_LSt
Co_C8_LF
Co_C9_BV
Co_C10_BCE
Co_C11_MEC
Co_C12_FCEC
Co_C13_IC
Co_C14_LV
Identity
Features
KRT13 S100A8 KRT6A PAX6 TP63 MITF CPVL GPHA2 KERA MMP3 FBLN1 CCL3 CCL5 ACTA2 POSTN MLANA
Percent Expressed
Average Expression
B
Ca_C1_ASK
Ca_C2_GSK
Ca_C3_GSK
Ca_C4_TSK
Ca_C5_BCE
Ca_C6_LCE
Ca_C7_WSEL
Ca_C8_LSC
Ca_C9_CEM
Ca_C10_CLC
Ca_C11_CES
Ca_C12_UN
Ca_C13_ESD
KERA LUM KRT12 KRT15 S100A2 S100A8 GJB2 GJB6 CAV1 CPVL CXCL14 KRT3 UBE2C CLDN7 LAMB2 CD109 COL4A3
C
Ga_C1_CF
Ga_C2_Cj
Ga_C3_THCEC
Ga_C4_EHCEC
Ga_C5_Cj
Ga_C6_Cj
Ga_C7_Cj
Ga_C8_Mel
Ga_C9_THCEC
Ga_C10_CF
Ga_C11_Mon
Ga_C12_CTC
KRT14 KRT19 HOPX ANXA1 EZR TGFBI SOX10 CYB561A3 CXCL2
D
Li_C1_DC
Li_C2_Cj
Li_C3_DC
Li_C4_PC
Li_C5_DC
Li_C6_PC
Li_C7_Mel
Li_C8_TALSC
Li_C9_LSC
Li_C10_UN
KRT12 KRT3 GJA1 MKI67 BIRC5 RRM2 KRT14 KRT15 KRT19 POSTN A2M TYR TYRP1 MLANA KRT4 KRT7 KRT13

Fig. S2. Defining cell states in the human cornea across individual corneal studies based on proposed marker genes. UMAPs of defined cell states and dot plots of marker genes in the study of (A) Collin (B) Català (C) Gautam (D) Li. The original identified cell states in individual studies are listed below.

Names of cell states (A): BCE= Basal corneal epithelium; LNCP = Limbal neural crest-derived progenitor cells; CjB= Conjunctival basal; SCE = Superficial corneal epithelium; LV = Lymphatic vessels; LPC = Limbal progenitor cells; CjS = Conjunctival superficial; LSt = Limbal stromal cells; CSSC = Corneal stromal stem cells; LF = Limbal fibroblasts; FCEC = Fibroblast corneal endothelial cells; BV = Blood vessels; IC = Immune cells; MEC = Melanocytes and endothelial cells.

Names of cell states (B): LSC = Limbal stem cells; LCE = Limbal corneal epithelium; WSEL = Wing superficial epithelial limbal cells; BCE= Basal corneal epithelium; CLC = Corneal limbal cells; TSK = Transitioning stromal keratocytes; ASK = activated stromal keratocytes; CEM= Corneal endothelium migratory; UN = Uncharacterizable; CES = Corneal endothelium stationary; GSK = General stroma keratocytes; ESD = Epidermal-stromal doublets.

Names of cell states (C): CTC = Cytotoxic T-cells; Mon = Monocytes; THCEC = TGFβ-high corneal epithelial cells; EHCEC = ELF3-high corneal epithelial cells; Cj= Conjunctiva; CF = Corneal fibroblasts; Mel = Melanocytes.

Names of cell states (D): DC = Differentiated cells; PC = Progenitor cells; Cj= Conjunctiva; UN = Uncharacterizable; LSC = Limbal stem cells; TALSC = Transient amplifying limbal stem cells; Mel = Melanocytes.

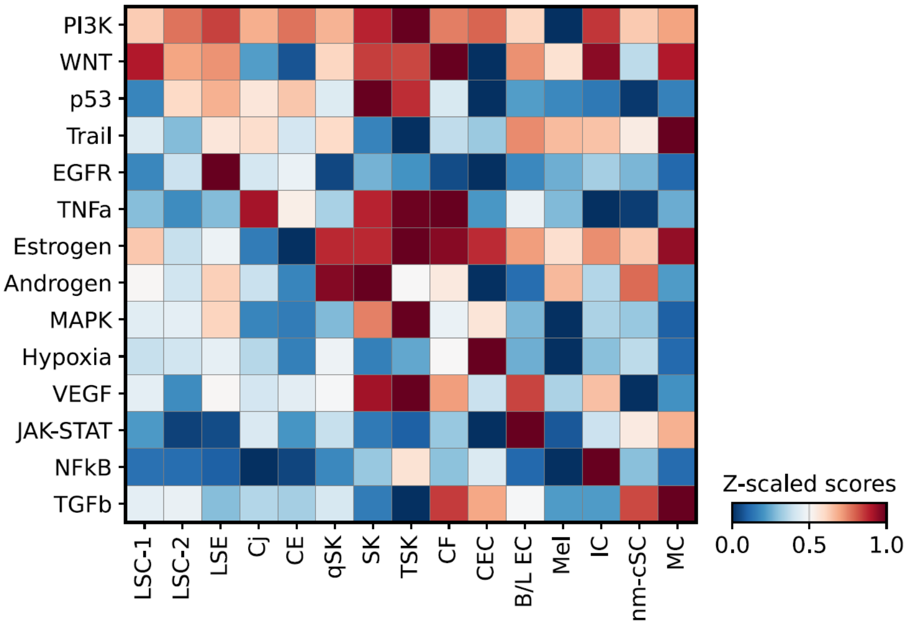

Fig. S3. Pathway analysis of cell states in the corneal meta-atlas with PROGENy. PROGENy scores scaled between 0 and 1 for 14 curated pathways based on the expression of genes involved in the respective pathways.

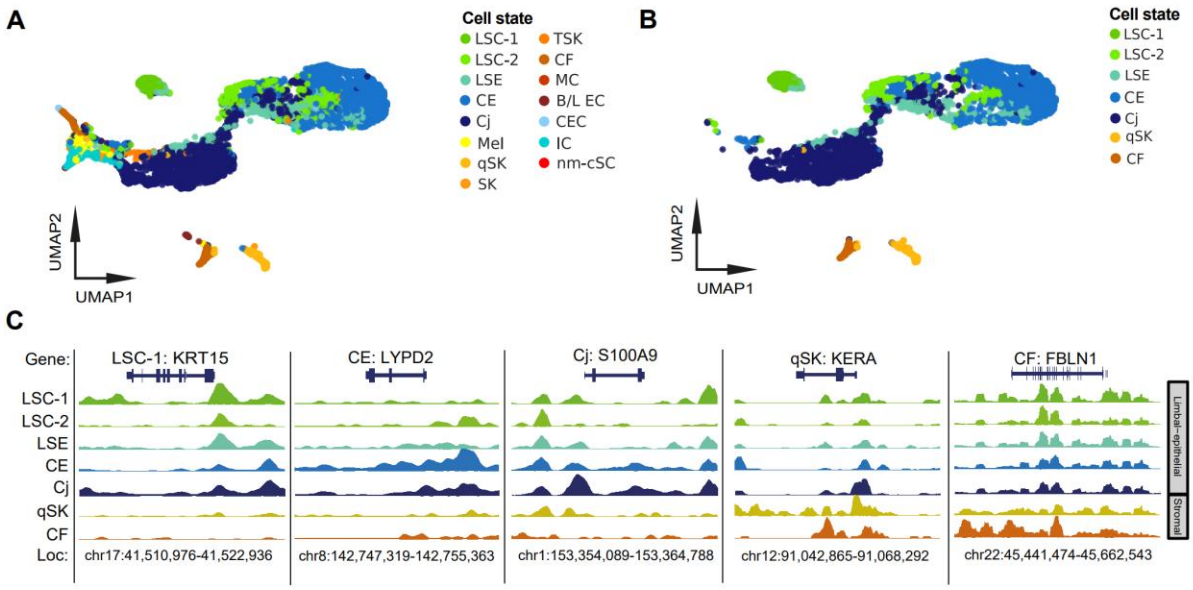

Fig. S4. Characterization of scATAC-seq signals across cell states in the human cornea. (A) UMAP of scATAC-seq imputed from scRNA-seq using Seurat label transfer. (B) UMAP of sub selected cell states with at least 100 cells and a minimal prediction score of 0.4. (C) Genome browser tracks showing open chromatin profiles around cell state specific marker genes.

Names of cell states: LSC-1 = Limbal stem cells 1; LSC-2 = Limbal stem cells 2; LSE = Limbal suprabasal epithelium; CE = Central epithelium; Cj = Conjunctiva; Mel = Melanocytes
CEC = Corneal endothelial cells; IC = Immune cells; B/L EC = Blood and lymph endothelial cells; qSK = Quiescent stromal keratocytes; SK = Stromal keratocytes; TSK = Transitioning stromal keratocytes; CF = Corneal fibroblasts; nm-cSC = non-myelinating corneal Schwann cells

A

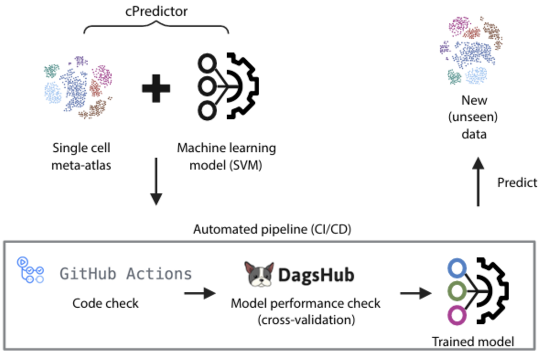

B

	precision	recall	f1-score	number of cells
LSC-1	0.94	0.96	0.96	4275
LSC-2	0.93	0.95	0.94	6643
LSE	0.98	0.97	0.97	11278
Cj	0.94	0.94	0.94	11955
CE	0.93	0.93	0.93	10908
qSK	0.97	0.98	0.98	5646
SK	0.97	0.97	0.97	4103
TSK	0.97	1.00	0.98	1414
CF	0.99	0.99	0.99	5585
CEC	1.00	1.00	1.00	813
B/L EC	0.99	0.98	0.98	688
Mel	0.99	0.97	0.98	827
IC	0.99	0.94	0.96	523
nm-cSC	1.00	0.99	0.99	81
MC	0.97	1.00	0.99	297
weighted avg	0.96	0.96	0.96	65036

C

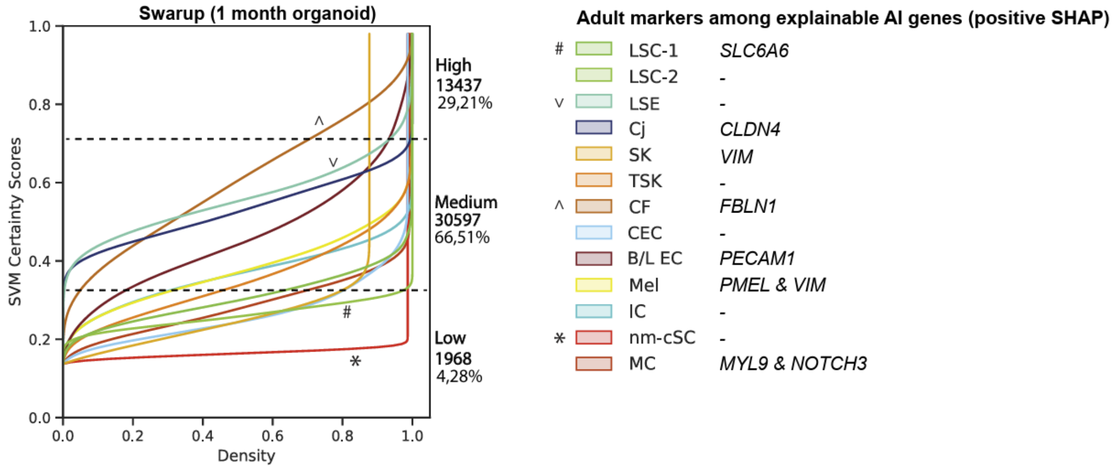

D

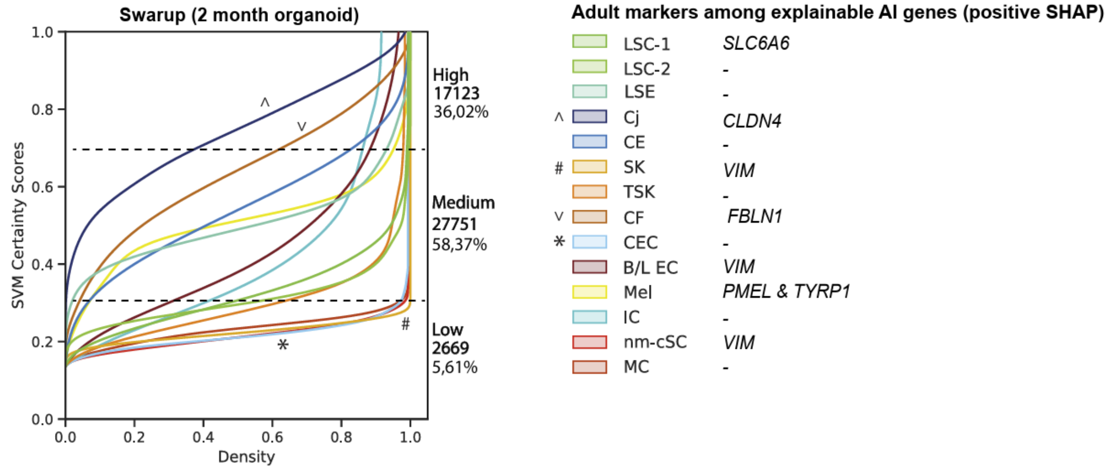

E

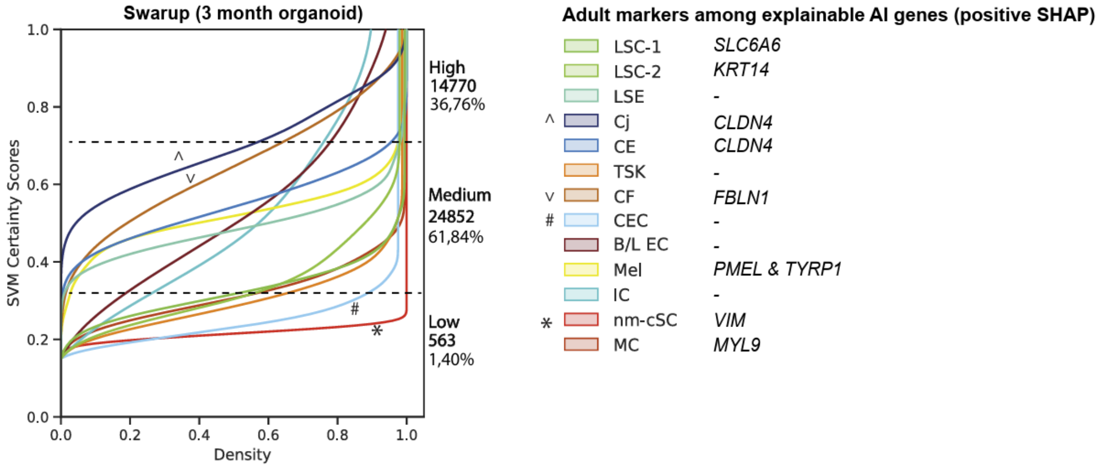

Fig. S5. Machine learning-based predictions of human corneal cell states in scRNA-seq datasets. (A) Schematic overview of cPredictor's machine learning pipeline. cPredictor applies the human corneal meta-atlas as reference data to support vector machines (SVM), which enables annotation of adult corneal cell states in external datasets. B) Sklearn's (16) classification report on the performance of a five-fold cross-validation using the cornea cell state meta-atlas. (C-E) Prediction certainty plots of corneal cell states on 1-month-old (C), 2-month-old (D) and 3-month-old organoids from Swarup (7) (E). Left, the x-axis shows the cumulative kernel densities, and the y-axis depicts the model confidence (SVM certainty score). The numbers and percentages of cells corresponding to low (<0.3), medium (>0.3 and <0.7) and high (>0.7) certainty scores in each dataset are depicted next to the plots. ^/v indicate cell states most similar and */# indicate cell states least similar to cell states from the corneal meta-atlas. Right, corneal markers among the top ten explainable AI genes driving model's decisions for each of the adult corneal cell states determined by their SHAP positive values are shown.

A

	gene 1	gene 2	gene 3	gene 4	gene 5	gene 6	gene 7	gene 8	gene 9	gene 10
LSC-1	MT1X	**KRT14**	**SLC6A6**	**S100A2**	DST	**KRT15**	PMAIP1	MIR205HG	ID3	**GPHA2**
LSC-2	ALDH3A1	IGFBP6	**KRT14**	NQO1	MIR205HG	TGFBI	HTRA1	TKT	CAMK2N1	COL17A1
LSE	RPS10	FABP5	TNFRSF12A	HMGA1	**AREG**	SFN	SH3BGRL3	**KRT14**	SOD2	FOSL1
Cj	**CLDN4**	AQP3	PTGS2	PERP	TGFBI	TACSTD2	BTG1	S100A11	NEAT1	CD55
CE	**KRT3**	CD24	ADIRF	PTGDS	**KRT12**	**CLDN4**	APOBEC3A	FABP5	PERP	UPK3BL1
qSK	ANGPTL7	RPS4Y1	**LUM**	**KERA**	HMOX1	HTRA1	DDX3Y	STEAP4	PTGDS	CD81
SK	DCN	SAA1	ITGBL1	CD81	MRPS24	FOSL1	**VIM**	TNFRSF12A	**MMP3**	IFITM2
TSK	RPS4Y1	MT1X	PTX3	MT2A	SSR3	DDX3Y	SERPINA3	DCN	LUCAT1	MYDGF
CF	CXCL1	NNMT	IGFBP5	SAA1	**FBLN1**	COL12A1	HTRA1	IGFBP7	RND3	IGFBP4
CEC	MGP	PTGDS	**SLC4A11**	COL4A3	ENO1	SFRP1	ITM2C	CCDC144A	APP	CA12
B/L EC	CLDN5	GNG11	**VIM**	**ACKR1**	**PECAM1**	TGFBR2	KLF2	TCF4	CCL14	SPARCL1
Mel	DCT	**PMEL**	**TYRP1**	**MLANA**	APOE	QPCT	**VIM**	CD63	MITF	TRPM1
IC	SRGN	**CCL4**	**CCL3**	CYBA	**VIM**	STK4	CCL3L1	BTG1	DUSP2	HLA-B
nm-cSC	**CDH19**	GPM6B	NRXN1	**SCN7A**	LGI4	KCNMB4	RPS4Y1	PLP1	SAMHD1	WNT6
MC	TAGLN	ID4	**MYL9**	NR2F2	C11orf96	RGS5	**NOTCH3**	SPARCL1	TPM1	PPP1R12A

B

	gene 1	gene 2	gene 3	gene 4	gene 5	gene 6	gene 7	gene 8	gene 9	gene 10
LSC-1	**S100A2**	MT1X	MIR205HG	KRT17	DST	ID3	MT-CO3	**KRT15**	SNHG6	SNHG29
LSC-2	IGFBP6	**BCAM**	MIR205HG	STMN1	ANXA1	CAMK2N1	TKT	KRT5	IER3	PLEC
LSE	RPS10	TNFRSF12A	GAPDH	SFN	SH3BGRL3	FABP5	KRT6A	HMGN3	HMGA1	SRSF5
Cj	AQP3	S100A11	**KRT13**	**CLDN4**	BTG1	NEAT1	PERP	KRT17	ITGA2	DSC2
CE	CD24	KRT17	S100A14	KRT5	ID1	TKT	S100A9	KRT13	FTH1	IGFBP2
SK	**VIM**	ANXA5	ANXA1	EIF5A	TNFRSF12A	MRPS24	SNHG7	KLF6	RPS4X	CEBPD
TSK	MT1G	MT-CYB	MT1X	MT-ND2	SNHG29	SSR3	NUDC	CCDC85B	MTDH	CCNI
CF	TIMP1	**VIM**	**FBLN1**	KRT17	RND3	FTL	SNHG5	EIF1B	AKAP12	CYP1B1
CEC	TTN	ENO1	PTGDS	**TNNC1**	IER3	NFKBIA	ID1	MT-CO1	APOE	APP
B/L EC	CLDN5	**VIM**	GNG11	CRIP2	ADGRL4	TCF4	ARHGAP29	EGFL7	**PECAM1**	HLA-E
Mel	DCT	**PMEL**	APOE	**VIM**	**TYRP1**	CD63	SDCBP	CD59	KLF6	STMN1
IC	SRGN	**CCL4**	**CCL3**	PTPRC	LAPTM5	STK4	B2M	SAMSN1	CYBA	CD44
nm-cSC	**VIM**	CALM2	WSB1	CRYAB	GPM6B	HSP90AB1	NRXN1	CST3	CNN3	RHOB
MC	**MYL9**	A2M	TAGLN	TPM1	CALD1	PPP1R12A	**ACTA2**	SEPTIN7	ID4	DSTN

C

	gene 1	gene 2	gene 3	gene 4	gene 5	gene 6	gene 7	gene 8	gene 9	gene 10
LSC-1	MT1X	**KRT15**	**SLC6A6**	**KRT14**	TXNIP	DST	**S100A2**	**GPHA2**	ID3	ATF3
LSC-2	ALDH3A1	**BCAM**	**KRT14**	STMN1	IGFBP6	FTH1	NQO1	TKT	ANXA1	**KRT12**
LSE	RPS10	GAPDH	SFN	S100A10	TNFRSF12A	HMGN3	SH3BGRL3	FABP5	FOSL1	CST3
Cj	S100A11	**KRT13**	**CLDN4**	AQP3	KRT19	CD55	**KRT15**	MGST1	BAG1	FTH1
CE	CD24	S100A14	KRT17	ID1	PTGDS	MT1X	MGARP	ELF3	COX7B	SLC20A1
SK	RPS4X	**VIM**	ANXA5	EIF5A	IFITM2	ANXA1	HSPD1	HSPB1	S100A10	TNFRSF12A
TSK	MT1G	MT1X	MT2A	NOP53	MT1E	EEF1D	RPS10	SSR3	PTGDS	CCDC85B
CF	**FBLN1**	TIMP1	SELENOM	LGALS1	EIF1B	FTL	LRP1	CKB	IFITM3	IGFBP5
CEC	PTGDS	ENO1	GAPDH	NFKBIA	IRF1	ITM2C	NDUFA1	TPI1	ZFHX3	IER3
B/L EC	GNG11	EGFL7	**VIM**	TGFBR2	TFPI	CRIP2	CTSH	CD74	ARHGAP29	MGST2
Mel	DCT	APOE	**PMEL**	**TYRP1**	GPX3	**VIM**	QPCT	CD63	CD59	MT1G
IC	CYBA	B2M	STK4	SRGN	ZFP36L2	PTPRC	WIPF1	CXCR4	CREM	HSPD1
MC	TAGLN	**MYL9**	PTMA	NR2F2	DSTN	CALD1	TPM1	PPP1R12A	SEPTIN7	IFITM3

D

	gene 1	gene 2	gene 3	gene 4	gene 5	gene 6	gene 7	gene 8	gene 9	gene 10
LSC-1	MT1X	DST	**SLC6A6**	NOP53	RPS3A	RPS27	PTMA	RPS2	EZR	ID3
LSC-2	STMN1	TKT	SAT1	RPS4X	MT1X	RPL5	SLC38A2	ANP32B	RPS18	RPL37A
LSE	RPS10	HMGA1	GAPDH	FABP5	GJA1	S100A10	HMGN3	SLC25A6	RPS8	BZW1
Cj	S100A11	BTG1	MGST1	SAT1	**CLDN4**	PRDX1	FTH1	KRT19	TMSB4X	SLC2A1
SK	RPS4X	HSPD1	EIF5A	ANXA5	**VIM**	EIF1AX	IFITM2	HSP90AB1	RPS27	SLC2A1
TSK	MT1G	MT1X	NOP53	MT1E	SSR3	DHX36	EEF1D	NUDC	MT2A	CCNI
CF	**FBLN1**	TIMP1	EIF1B	COL3A1	FTL	**COL1A1**	AMD1	NPM1	COL1A2	IFITM3
CEC	ENO1	ZFHX3	APOE	APP	NDUFA1	TMEM160	ATP5ME	FUS	EPB41L2	UQCR11
B/L EC	KDR	TFPI	MMRN1	GNG11	CALCRL	EGFL7	LDB2	**PECAM1**	ARHGAP29	MEF2C
Mel	DCT	**PMEL**	APOE	EDNRB	CD63	STMN1	**VIM**	MYO5A	RBM3	CHCHD6
IC	HSPD1	CYBA	ANKRD28	B2M	ZEB2	ZFP36L2	DHX36	AKAP13	STK4	LCP1
nm-cSC	GPM6B	CALM2	NRXN1	SAMHD1	WSB1	KCNMB4	WNT6	HSP90AB1	MT1G	CHPT1
MC	PPP1R12A	SEPTIN7	CALD1	DSTN	TPM1	NR2F2	ID4	PTMA	**NOTCH3**	**MYL9**

E

	gene 1	gene 2	gene 3	gene 4	gene 5	gene 6	gene 7	gene 8	gene 9	gene 10
LSC-1	DST	**SLC6A6**	RPS3A	RPS27	MT1X	NOP53	RPL5	RPL13	BTF3	RPL10
LSC-2	SAT1	STMN1	FTH1	CREB5	TKT	ANXA1	CSRP2	KCNMA1	BTG1	SLC38A2
LSE	GAPDH	RPS10	RPS8	SH3BGRL3	KRT17	TNFRSF12A	UBC	RPS9	RPS27	S100A10
Cj	S100A11	**CLDN4**	SAT1	KRT19	BTG1	FTH1	TMSB4X	CD55	PERP	AQP3
CE	CD24	FTH1	B2M	ELP4	IGFBP2	ELF3	TKT	C4orf3	CALM2	RPL3
SK	RPS4X	**VIM**	RPS8	RPS18	HSP90AB1	ANXA5	ANXA1	RPS2	HSPD1	RPL10
TSK	EEF1D	NOP53	RPL13	RPLP0	CCNI	RPS10	SSR3	MTDH	AHI1	EEF1A1
CF	TIMP1	**FBLN1**	IGFBP5	FTL	RPS4X	RPS9	EIF1B	CKB	DCT	HLA-E
CEC	ENO1	ZFHX3	GAPDH	PTGDS	FTL	PCDH7	APP	MIF	SFRP1	NDUFA1
B/L EC	ARHGAP29	**VIM**	TFPI	TCF4	FTH1	EGFL7	ZNF385D	MARCKSL1	HLA-E	TMSB10
Mel	DCT	**PMEL**	**TYRP1**	APOE	IGFBP5	CD63	TRPM1	TIMP1	**VIM**	MITF
IC	CXCR4	PTPRC	B2M	CYBA	STK4	ZEB2	WIPF1	ANKRD28	FTH1	EVI2B
nm-cSC	NRXN1	GPM6B	CALM2	**VIM**	SORBS2	WSB1	CHPT1	HSP90AB1	KCNMB4	CST3
MC	PPP1R12A	EBF1	NR2F2	SEPTIN7	PTMA	CALD1	DSTN	TPM1	ZEB2	LPP

F

	gene 1	gene 2	gene 3	gene 4	gene 5	gene 6	gene 7	gene 8	gene 9	gene 10
LSC-1	MT1X	DST	NOP53	RPS27	RPS3A	**SLC6A6**	TXNIP	WFDC2	BTF3	ID3
LSC-2	ANXA1	STMN1	FTH1	TKT	ALDH3A1	SAT1	**KRT14**	GLUL	ANP32B	NPM1
LSE	RPS10	GAPDH	RPS8	TNFRSF12A	SH3BGRL3	CST3	S100A10	HMGN3	SFN	SLC25A6
Cj	S100A11	BTG1	**CLDN4**	KRT19	SAT1	MT1X	FTH1	VEGFA	BAG1	PERP
CE	CD24	S100A11	MT1X	TKT	S100A14	**CLDN4**	KRT17	PERP	ELF3	**LYPD2**
TSK	MT1X	NOP53	EEF1D	CCNI	SSR3	MTDH	AHI1	RPLP0	NUDC	RPS10
CF	TIMP1	**FBLN1**	IGFBP5	FTL	EIF1B	CKB	PLIN2	**VIM**	SELENOM	ZFP36L2
CEC	PTGDS	ENO1	ZFHX3	GAPDH	PCDH7	APP	TSPYL2	DDB1	MIF	NDUFA1
B/L EC	TFPI	EGFL7	ARHGAP29	TGFBR2	HYAL2	MGST2	TCF4	CD59	**VIM**	MARCKSL1
Mel	DCT	**PMEL**	**TYRP1**	APOE	CD63	CD59	QPCT	**VIM**	SDCBP	CHCHD6
IC	EVI2B	PTPRC	CYBA	B2M	STK4	STK17B	HLA-B	LCP1	ZFP36L2	HLA-C
nm-cSC	GPM6B	NRXN1	**VIM**	CALM2	CST3	KCNMB4	WSB1	CHPT1	SORBS2	HSP90AB1
MC	PPP1R12A	SEPTIN7	DSTN	PTMA	CALD1	NR2F2	ID4	**MYL9**	TIMP3	TPM1

Fig. S6. Top positively contributing explainable AI genes with SHAP analysis. (A-F) Top 10 explainable genes positively contributing to model decisions for Maiti data (A-B): from adult cornea (A) and from 4-month organoids (B), and for Swarup data (C-F): 4-month organoids (C), 1-month organoids (D), 2-month organoids (E), 3-month organoids (F). Adult markers for cell states are highlighted in bold.

Table S1. Description of experimental setup of the four studies and associated datasets used for construction of the corneal cell state meta-atlas.

Study	Number of donor corneas	Sample disaggregation method	Specifically retrieved regions	Number of analysed single cells	Additional data
Collin et al.	6	Enzymatic digestion of corneas	Limbal ring (1), central cornea (1), complete corneas (4)	22276	scATAC-seq
Català et al.	8	Dissection of specific corneal regions	Limbal ring (2), complete corneas (6)	15903	-
Gautam et al.	3	Dissection of eye tissue parts	Whole eyes with cornea data included (3)	10248	-
Li et al.	4	Dissection of the limbal region	Limbus after removing central cornea and superficial layers (4)	16609	-

Table S2. Antibodies used in this study.

Primary antibodies	Host	Company	Catalogue Number	Dilution
CPVL	Rabbit polyclonal	Thermo-Fisher	PA5-63308	1:100
SLC6A6	Rabbit polyclonal	Fisher Scientific	16500992	1:100
p63 (ΔNp63)	Mouse monoclonal [4A4]	Abcam	ab735	1:100
Keratocan	Rabbit polyclonal	Sigma-Aldrich	HPA039321	1:100
Fibulin-1	Rabbit polyclonal	Fisher Scientific	16620295	1:100
POU3F3	Rabbit polyclonal	Abcam	ab247159	1:100
TNNC1	Mouse polyclonal [4C2]	Abcam	ab10231	1:100

SI references

1. van der Sande M, Frölich S, Schäfers T, Smits JGA, Snabel RR, Rinzema S, et al. Seq2science: an end-to-end workflow for functional genomics analysis. PeerJ. 2023 Nov 15;11:e16380.

2. Zheng GXY, Terry JM, Belgrader P, Ryvkin P, Bent ZW, Wilson R, et al. Massively parallel digital transcriptional profiling of single cells. Nat Commun. 2017 Jan 16;8(1):14049.

3. Satpathy AT, Granja JM, Yost KE, Qi Y, Meschi F, McDermott GP, et al. Massively parallel single-cell chromatin landscapes of human immune cell development and intratumoral T cell exhaustion. Nat Biotechnol. 2019 Aug;37(8):925–36.

4. Fang R, Preissl S, Li Y, Hou X, Lucero J, Wang X, et al. Comprehensive analysis of single cell ATAC-seq data with SnapATAC. Nat Commun. 2021 Feb 26;12(1):1337.

5. Stuart T, Butler A, Hoffman P, Hafemeister C, Papalexi E, Mauck WM, et al. Comprehensive integration of single-cell data. Cell. 2019 Jun 13;177(7):1888-1902.e21.

6. McGinnis CS, Murrow LM, Gartner ZJ. DoubletFinder: Doublet Detection in Single-Cell RNA Sequencing Data Using Artificial Nearest Neighbors. Cell Syst. 2019 Apr 24;8(4):329-337.e4.

7. Swarup A, Phansalkar R, Morri M, Agarwal A, Subramaniam V, Li B, et al. Single-cell transcriptomic analysis of corneal organoids during development. Stem Cell Reports. 2023 Dec 12;18(12):2482.

8. Zappia L, Oshlack A. Clustering trees: a visualization for evaluating clusterings at multiple resolutions. GigaScience. 2018 Jul 1;7(7):giy083.

9. Traag VA, Waltman L, van Eck NJ. From Louvain to Leiden: guaranteeing well-connected communities. Sci Rep. 2019 Mar 26;9(1):5233.

10. Svensson V, Gayoso A, Yosef N, Pachter L. Interpretable factor models of single-cell RNA-seq via variational autoencoders. Bioinformatics. 2020 Jun;36(11):3418–21.

11. Kamil Slowikowski, John Arevalo, Jonathan Manning. slowkow/harmonypy: harmonypy version 0.0.10. Zenodo; 2024.

12. Carlson M. org.Hs.eg.db: Genome wide annotation for Human. R package version 3.4.1 (2017).

13. Benjamini Y, Hochberg Y. On the Adaptive Control of the False Discovery Rate in Multiple Testing With Independent Statistics. Journal of Educational and Behavioral Statistics. 2000 Mar 1;25(1):60–83.

14. Schubert M, Klinger B, Klünemann M, Sieber A, Uhlitz F, Sauer S, et al. Perturbation-response genes reveal signaling footprints in cancer gene expression. Nat Commun. 2018 Jan 2;9:20.

15. J Arts. Arts-of-coding/cPredictor: v0.5.0. Zenodo; 2025.

16. Pedregosa F, Varoquaux G, Gramfort A, Michel V, Thirion B, Grisel O, et al. Scikit-learn: Machine Learning in Python. Journal of Machine Learning Research. 2011;12(85):2825–30.

17. O'Connor PDT, Kleyner A. Monte Carlo Simulation. In: Practical Reliability Engineering [Internet]. John Wiley & Sons, Ltd; 2011 [cited 2024 Oct 15]. p. 108–19. Available from: https://doi.org/10.1002/9781119961260.ch4

18. Maiti G, Monteiro de Barros MR, Hu N, Dolgalev I, Roshan M, Foster JW, et al. Single cell RNA-seq of human cornea organoids identifies cell fates of a developing immature cornea. Levine B, editor. PNAS Nexus. 2022 Nov 1;1(5):pgac246.

19. Zhang Y, Liu T, Meyer CA, Eeckhoute J, Johnson DS, Bernstein BE, et al. Model-based Analysis of ChIP-Seq (MACS). Genome Biology. 2008 Sep 17;9(9):R137.

20. Bruse N, Heeringen SJ van. GimmeMotifs: an analysis framework for transcription factor motif analysis. bioRxiv. 2018 Jan 1;474403.

21. Li Z, Schulz MH, Look T, Begemann M, Zenke M, Costa IG. Identification of transcription factor binding sites using ATAC-seq. Genome Biol. 2019 Feb 26;20:45.

22. Smits JGA, Arts JA, Frölich S, Snabel RR, Heuts BMH, Martens JHA, et al. scANANSE gene regulatory network and motif analysis of single-cell clusters. F1000Res. 2023;12:243.

23. Taubenschmid-Stowers J, Rostovskaya M, Santos F, Ljung S, Argelaguet R, Krueger F, et al. 8C-like cells capture the human zygotic genome activation program in vitro. Cell Stem Cell. 2022 Mar 3;29(3):449-459.e6.
